# ZNF506-dependent positive feedback loop regulates H2AX signaling after DNA damage

**DOI:** 10.1038/s41467-018-05161-0

**Published:** 2018-07-16

**Authors:** Somaira Nowsheen, Khaled Aziz, Kuntian Luo, Min Deng, Bo Qin, Jian Yuan, Karthik B. Jeganathan, Jia Yu, Henan Zhang, Wei Ding, Jan M. van Deursen, Zhenkun Lou

**Affiliations:** 1 Mayo Clinic Medical Scientist Training Program, Mayo Clinic School of Medicine and Mayo Clinic Graduate School of Biomedical Sciences, Mayo Clinic, Rochester, MN 55905 USA; 2 Department of Molecular Pharmacology and Experimental Therapeutics, Mayo Clinic, Rochester, MN 55905 USA; 3 Department of Oncology, Mayo Clinic, Rochester, MN 55905 USA; 4 Research Center for Translational Medicine, Key Laboratory of Arrhythmias of the Ministry of Education of China, East Hospital, Tongji University School of Medicine, Shanghai, 200120 China; 5 Department of Pediatrics and Adolescent Medicine, Mayo Clinic, Rochester, MN 55905 USA; 6 Department of Hematology, Mayo Clinic, Rochester, MN 55905 USA

## Abstract

Cells respond to cytotoxic DNA double-strand breaks by recruiting repair proteins to the damaged site. Phosphorylation of the histone variant H2AX at S139 and Y142 modulate its interaction with downstream DNA repair proteins and their recruitment to DNA lesions. Here we report ATM-dependent ZNF506 localization to the lesion through MDC1 following DNA damage. ZNF506, in turn, recruits the protein phosphatase EYA, resulting in dephosphorylation of H2AX at Y142, which further facilitates the recruitment of MDC1 and other downstream repair factors. Thus, ZNF506 regulates the early dynamic signaling in the DNA damage response (DDR) pathway and controls progressive downstream signal amplification. Cells lacking ZNF506 or harboring mutations found in cancer patient samples are more sensitive to radiation, offering a potential new therapeutic option for cancers with mutations in this pathway. Taken together, these results demonstrate how the DDR pathway is orchestrated by ZNF506 to maintain genomic integrity.

## Abstract (web summary)

Following double-strand break a cascade of events leads to the recruitment of repair factors to damaged sites. Here the authors identify ZNF506 as a key factor that mediates post-translational modification changes in H2AX affecting the DNA damage response.

## Introduction

Our genome is under constant stress, both from exogenous and endogenous agents that can lead to various forms of DNA damage. Of the various types of DNA damage, DNA double-strand breaks are the most lethal. Since one unrepaired DNA double-strand break can potentially be lethal to the cell, cells have evolved an intricate system called the DNA damage response system to combat this threat and maintain genomic integrity^1–3^. This response to cytotoxic DNA double-strand breaks involves accrual of DNA repair proteins to the damaged site. This complex response system starts with the protein kinase ATM sensing the damaged DNA and phosphorylating the histone variant H2AX at its serine 139 (Ser139) residue, thereby forming γ-H2AX^4^. γ-H2AX is required for subsequent interaction with downstream DNA repair proteins^5,6^.

The mediator protein MDC1 interacts with γ-H2AX using its BRCT domain^7,8^. However, before H2AX can recruit MDC1 via its phosphorylated S139 site, it has to be dephosphorylated at Y142 by the phosphatase EYA^5,6^. After MDC1 recruitment, MDC1 in turn recruits the E3 ligase RNF8, which ubiquitylates the polycomb group-like protein L3MBTL2^9^. Next, another E3 ligase, RNF168, recognizes the ubiquitylated L3MBTL2 and localizes to the sites of DNA damage^9^. This protein, RNF168, ubiquitylates lysine residues on histones H2A and H2AX (K13 and K15 residues) and further amplifies the response^10^. These ubiquitylated histone residues are recognized by DNA repair proteins such as 53BP1 and RAP80, which localize to the damage site to amplify and promote DNA double-strand break repair^11–16^.

As mentioned above, DNA repair is very complex with significant cross-talk between the various pathways. The DNA double-strand break response can be simplified to two major repair pathways: homologous recombination and non-homologous end joining. Homologous recombination-mediated DNA double-strand break repair is restricted to the S/G2 phase of the cell cycle since it requires the sister chromatid as a template for high-fidelity repair. Among others, BRCA1, BRCA2, and Rad51 are critical for efficient homologous recombination-mediated DNA double-strand break repair^17–19^. In contrast, non-homologous end-joining-mediated DNA double-strand break repair can occur throughout the cell cycle and is error prone. Key players in this pathway include proteins such as DNA-PK/Ku complex, 53BP1, Artemis, and Ligase IV. Regulators such as Rev7, TIRR, UHRF1, and Rif1 dictate the choice of DNA repair pathway^20–24^.

As DNA double-strand break repair pathways are often aberrant in cancers such as leukemia, identifying key regulators of this pathway is important for ascertaining novel therapeutic targets. In order to identify new regulators of this pathway, we analyzed the sequencing data of patients with a rare type of leukemia called T-cell prolymphocytic leukemia (T-PLL) in the Mayo Clinic patient database. We identified ZNF506 as one of the frequently mutated genes in this patient population. To our knowledge, there are no reports on ZNF506 in any biological context; hence, we proceeded to identify the function of this protein.

In this study, we reveal that ZNF506 regulates gradual H2AX dephosphorylation at Y142. We report that DNA damage-induced ATM-dependent phosphorylation of ZNF506 localizes it to the DNA damage site through its interaction with MDC1. ZNF506, in turn, facilitates recruitment of the protein phosphatase EYA to the DNA lesion, dephosphorylating H2AX at Y142 and leading to recruitment of MDC1 and other downstream repair factors. In this way, ZNF506 regulates the dynamics at an early point in the DNA damage response pathway and controls progressive downstream signal amplification. Overexpression of ZNF506 confers resistance to radiation. Conversely, cells lacking ZNF506 or harboring mutations found in patient samples are more sensitive to radiation and DNA-damaging agents, offering a potential new therapeutic option for cancers with mutations in this pathway. Collectively, these results identify ZNF506 as a key target of ATM following DNA damage and establish the function of ZNF506 in maintaining genomic integrity through its role in the DNA damage response pathway.

## Results

### ZNF506 localizes to DNA lesions in an MDC1-dependent manner

**Fig. 1:**
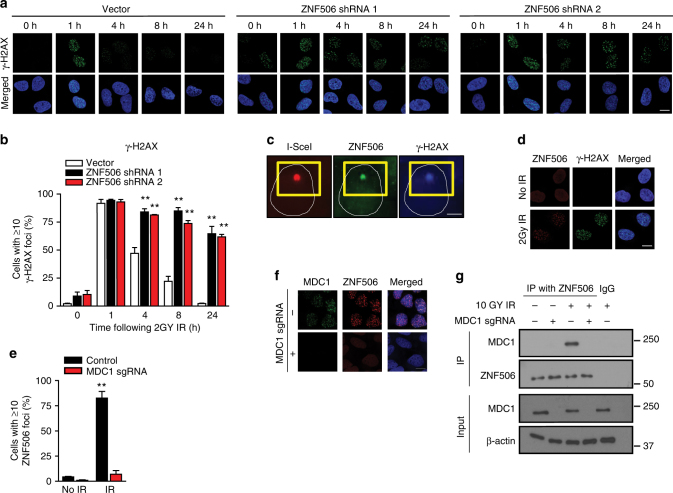
ZNF506 localizes to DNA double-strand break in an MDC1-dependent manner. **a**, **b** Downregulation of ZNF506 using shRNAs leads to persistent DNA double-strand breaks. **a** Representative images of γ-H2AX foci (green) in U2OS cells after the indicated treatments and indicated time following 2 Gy irradiation (IR). Nucleus is stained with DAPI (blue). **b** Quantification of γ-H2AX foci in U2OS cells after the indicated treatment and time following IR. **c** ZNF506 localizes to the DNA double-strand break site in U2OS I-SceI cells where one DNA double-strand break is induced per cell using triamcinolone acetonide (red). This ZNF506 focus (green) overlaps with γH2AX (blue). The yellow box locates the site of the cut. The white circle outlines the nucleus. **d** ZNF506 forms radiation-induced puncta that overlaps with γ-H2AX in U2OS cells. Cells were stained for γ-H2AX foci (green). Nucleus is stained with DAPI (blue). Endogenous ZNF506 shows diffuse nuclear staining without DNA damage but forms discrete nuclear foci that overlaps with γ-H2AX upon treatment with 2 Gy IR (red). **e**, **f** MDC1 is required for recruitment of ZNF506 to double-strand break sites. U2OS cells in which MDC1 was knocked out using CRISPR were exposed to 2 Gy IR and stained for the indicated foci. Nucleus is stained with DAPI (blue). **e** Quantification of ZNF506 foci in U2OS cells wild-type or knockout for MDC1 and treated as indicated. **f** Representative images of the indicated foci in U2OS cells an hour after 2 Gy IR. **g** Endogenous ZNF506 interacts with MDC1 upon DNA damage. Shown are the representative data (mean ± SEM) from three independent experiments in **b** and **e**. **p < 0.01 by ANOVA comparing the knockdown groups to control at each time point in **b**, and control to knockout with each treatment condition in **e**. Representative images of three independent experiments are shown in **a**, **c**, **d**, and **f**. Scale bars, 10 µm. Representative western blots in **g** are provided from three biologically independent experiments. Unprocessed blots are provided in Supplementary Fig. 5

Since the gene was identified in T-PLL, a disease characterized by mutations in DNA double-strand break repair proteins such as ATM^25–27^, we tested whether ZNF506 plays a role in DNA double-strand break repair. We knocked down ZNF506 in U2OS cells using short hairpin RNAs (shRNAs) and assessed radiation-induced γ-H2AX foci, a marker for DNA double-strand breaks^4^. As shown in Fig. 1a, b, downregulation of ZNF506 resulted in increase in γ-H2AX signals in the absence of exogenous DNA damage, suggesting endogenous DNA damage in ZNF506-depleted cells. After ionizing radiation, ZNF506 depletion did not affect the initial formation of γ-H2AX foci but drastically attenuated the resolution of γ-H2AX foci, even 24 h following the initial DNA damage. Using the neutral comet assay (Supplementary Fig. 1a, b), we also found increased tail moments in ZNF506-depleted cells, suggesting that increased DNA damage is at least partly the cause for the increased and persistent γ-H2AX signals observed with ZNF506 depletion. Thus, DNA double-strand breaks persist in the absence of ZNF506 (Fig. 1a, b and Supplementary Fig. 1a–c). Next, we wanted to check if ZNF506 localizes to DNA double-strand break sites. We utilized an I-Sce1-based reporter system that induces one DNA double-strand break per cell^28^. As shown in Fig. 1c, ZNF506 localized to the site of DNA double-strand break, which overlapped with the γ-H2AX focus. This result was validated in U2OS cells where endogenous ZNF506 formed radiation-induced puncta that overlapped with γ-H2AX (Fig. 1d and Supplementary Fig. 1d). Taken together, our results suggest that ZNF506 localizes to the DNA double-strand break site following DNA damage and has functional implications in DNA damage response.

Next, we wanted to identify factor(s) required to retain ZNF506 at the damage site. We found that MDC1^29–31^, an important mediator of the DNA damage response pathway, is required for ZNF506 focus formation. In MDC1 knockout cells, ZNF506 localization to DNA double-strand break sites was significantly impaired (Fig. 1e, f and Supplementary Fig. 1e). Co-immunoprecipitation experiments in HEK293 cells irradiated to induce DNA damage revealed that ZNF506 interacts with MDC1 upon DNA damage (Fig. 1g). This puts forth the model that MDC1 is required for the retention of phosphorylated ZNF506 at DNA damage sites.

### ZNF506 is phosphorylated by ATM upon DNA damage

**Fig. 2:**
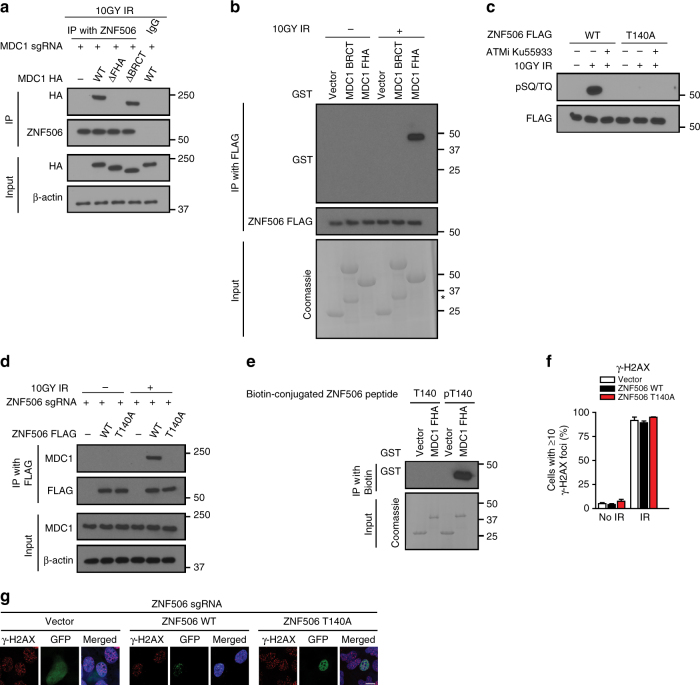
DNA damage-induced ATM-dependent phosphorylation of ZNF506 is required for its MDC1-mediated recruitment to the DNA double-strand break site. **a** ZNF506 interacts with the FHA domain of MDC1 upon DNA damage. Deletion of the FHA domain (ΔFHA) abrogates the interaction between MDC1 and ZNF506 in HEK293 cells. MDC1 knockout cells were transfected with the indicated plasmids and exposed to radiation. An hour later, interaction between endogenous ZNF506 and MDC1 fragments were assessed. **b** ZNF506 interacts with the FHA domain of MDC1 in vitro. **c** ATM phosphorylates ZNF506 at the T140 residue in HEK293 cells following DNA damage. ATM inhibitor (ATMi) KU55933 was used as a control. Cells were transfected with the indicated tagged constructs, co-immunoprecipitation was performed, and the blot was probed with pSQ/TQ antibody. **d** Phosphorylation of ZNF506 is required for its interaction with MDC1. Endogenous ZNF506 was knocked out in HEK293 cells using CRISPR and the indicated FLAG-tagged constructs were expressed. Cells were exposed to the indicated doses of radiation. Lysates were collected after an hour and immunoprecipitation with FLAG was performed. Blots were probed with the indicated antibodies. **e** The FHA domain of MDC1 directly interacts with phosphorylated ZNF506. The indicated peptides of ZNF506 (T140 (control) and pT140) were incubated with GST control vector or GST-tagged FHA domain of MDC1. The blot was probed with the indicated antibody. **f**, **g** Phosphorylation of ZNF506 is required for its recruitment to DNA double-strand break sites in U2OS cells. Mutation of the phosphorylation site on ZNF506 abrogates its localization to DSB sites. Cells were exposed to 2 Gy irradiation (IR) and stained for the indicated foci (red). Nucleus is stained with DAPI (blue). **f** Quantification of the indicated foci in U2OS cells after the indicated treatment. **g** Representative images of the indicated foci in U2OS cells an hour after 2 Gy IR. Shown are the representative data (mean ± SEM) from three independent experiments in **f**. Representative images of three independent experiments are shown in **g**. Scale bars, 10 µm. Representative western blots in **a**–**e** are provided from three biologically independent experiments. Unprocessed blots are provided in Supplementary Fig. 5

We next investigated how MDC1 helps retain ZNF506 at DNA damage sites. MDC1 contains tandem BRCT domains and a forkhead-associated (FHA) domain that recognizes phosphorylated serine/threonine-containing motifs^32–34^. We found that the MDC1-ZNF506 interaction was dependent on the FHA, but not BRCT domain of MDC1 both by co-immunoprecipitation and glutathione-*S*-transferase (GST) pull-down assays (Fig. 2a, b). Because the FHA domain recognizes phosphorylated motifs, we next hypothesized that ZNF506 might be phosphorylated. To assess if indeed ZNF506 is phosphorylated upon DNA damage, we expressed FLAG-tagged ZNF506 in HEK293 cells, exposed the cells to radiation, and performed co-immunoprecipitation experiments. As shown in Fig. 2c, probing the blots with pSQ/TQ antibody that recognizes putative ATM phosphorylation sites detected significant ZNF506 phosphorylation. Treatment with the ATM inhibitor Ku55933 attenuated this phosphorylation, suggesting that indeed ATM was the protein kinase responsible for this process. To identify the possible ATM phosphorylation sites on ZNF506, we analyzed the protein sequence for ATM phosphorylation sites which are typically pSQ/TQ^35^. As shown in Supplementary Fig. 2a, a possible threonine (T140) site was identified in ZNF506. Mutation of the site to alanine (T140A) abrogated DNA damage-induced ZNF506 phosphorylation (Fig. 2c). Taken together, this suggests that ZNF506 is phosphorylated by ATM at the T140 residue upon DNA damage.

Phosphorylation of ZNF506 was found to be critical for its interaction with MDC1 since no DNA damage-induced interaction between ZNF506 and MDC1 was detected in cells expressing the ZNF506 phosphorylation mutant T140A (Fig. 2d). Using purified GST-tagged FHA protein and either non-phosphorylated or phosphorylated ZNF506 peptides, we validated that this binding between ZNF506 and MDC1 is, in fact, a direct interaction (Fig. 2e). Collectively, these results establish that the FHA domain of MDC1 and phosphorylated T140 residue of ZNF506 mediate their interaction. Based on our results, one can predict that ZNF506 phosphorylation is required for its focus formation. Indeed, as shown in Fig. 2f, g, the phosphorylation mutant was unable to localize to sites of DNA damage as apparent from lack of radiation-induced foci, suggesting that ATM-mediated phosphorylation of ZNF506 is required for its interaction with MDC1 and its localization to the sites of DNA damage.

### ZNF506 recruits EYA for H2AX dephosphorylation at Y142

**Fig. 3:**
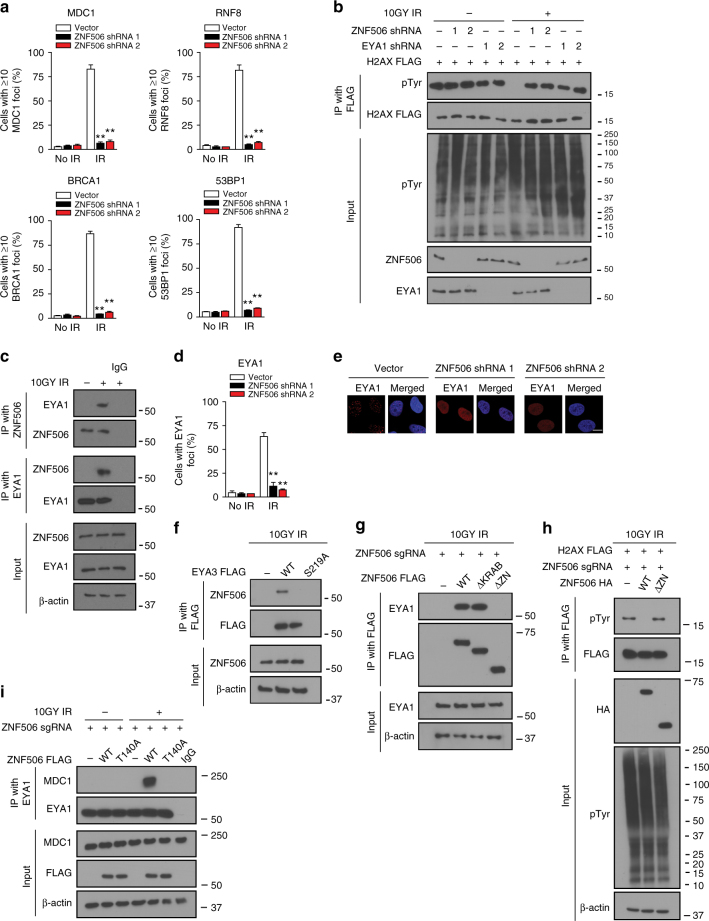
ZNF506 recruits EYA for H2AX dephosphorylation at Y142 upon DNA damage. **a** Knockdown of ZNF506 affects localization of DNA repair proteins to the DNA double-strand break site. **b** ZNF506 regulates H2AX dephosphorylation upon DNA damage. **c** DNA damage induces interaction between ZNF506 and EYA1. **d**, **e**, ZNF506 is required for the recruitment of EYA1 to DNA double-strand break sites. Cells were exposed to 2 Gy irradiation (IR) and stained for EYA1 focus formation (red). Nucleus is stained with DAPI (blue). **d** Quantification of EYA1 foci in U2OS cells after the indicated treatment. **e** Representative images of EYA1 foci in U2OS cells. **f** Phosphorylation of EYA3 is required for its interaction with ZNF506. Either wild-type (WT) or the phosphorylation mutant of EYA3 (S219A) were expressed in HEK293 cells. Cells were exposed to 10 Gy IR and co-immunoprecipitation was performed. **g** The zinc-finger domain of ZNF506 is critical for its interaction with EYA1. The indicated FLAG-tagged constructs of ZNF506 (WT: wild-type; ∆KRAB: deletion of KRAB domain; and ∆ZN: deletion of zinc-finger domains) were expressed in HEK293 cells in which the endogenous ZNF506 had been knocked out using CRISPR. Cells were exposed to 10 Gy IR. Lysates were collected after an hour, co-immunoprecipitation was performed, and blots were probed with the indicated antibodies. **h** The zinc-finger domain of ZNF506 is critical for its role in H2AX dephosphorylation at Y142. The indicated HA-tagged constructs of ZNF506 (WT: wild-type and ∆ZN: deletion of zinc-finger domains) and FLAG-tagged H2AX were expressed in HEK293 cells in which the endogenous ZNF506 had been knocked out using CRISPR. Co-immunoprecipitation was performed. **i** Phosphorylation of ZNF506 is required for the interaction between EYA1 and MDC1. Shown are the representative data (mean ± SEM) from three independent experiments in **a**, **d**. **p < 0.01 using ANOVA for vector vs. shRNA for each treatment condition (no IR or IR) in **a**, **d**. Representative images of three independent experiments are shown in **e**. Scale bars, 10 µm. Representative western blots in **b**, **c** and **f**- **i** are provided from three biologically independent experiments. Unprocessed blots are provided in Supplementary Fig. 5

The DNA double-strand break repair is a complex dynamic process with significant cross-talk between various players in the pathway. In order to assess the step(s) regulated by ZNF506, we systematically checked irradiation (IR)-induced foci formation of multiple DNA double-strand break repair proteins. The signaling cascade starts with phosphorylation of key substrates such as H2AX and MDC1 around the DNA double-strand break site by the protein kinase ATM. Phosphorylation of H2AX at S139 (γ-H2AX) recruits the mediator protein MDC1 to the double-strand break site. MDC1 in turn acts as an adaptor for signal amplification by recruiting proteins such as NBS1, RNF8, and so on. These proteins further amplify the signal and recruit downstream DNA repair proteins such as 53BP1 and BRCA1 to mediate efficient DNA double-strand break repair^36^. We systematically explored the focus formation of key DNA double-strand break repair proteins in ZNF506-depleted cells. Interestingly, our results revealed that ZNF506 regulates MDC1 accumulation at DNA double-strand break sites. As shown in Fig. 3a and Supplementary Fig. 3a, b, knockdown of ZNF506 attenuated the formation of radiation-induced MDC1 foci. Therefore, ZNF506 and MDC1 accumulation are dependent on each other, likely forming a positive feedback loop. As expected, the focus formation of DNA damage response factors downstream of MDC1, such as RNF8, BRCA1, and 53BP1, were also compromised in ZNF506-depleted cells. Since ZNF506 is a zinc-finger protein, we assessed if the effects observed were independent of transcription. Indeed, knockdown of ZNF506 using shRNAs did not affect the protein level of these repair factors (Supplementary Fig. 3a). This effect on the recruitment of DNA repair factors to sites of DNA damage was validated in ZNF506-knockout U2OS cells as well (Supplementary Fig. 3c–e). As further confirmation of the importance of wild-type ZNF506 in mediating this effect, radiation-induced foci formation of MDC1 and RNF8 was restored with the expression of wild-type but not the T140 phosphorylation mutant (T140A) of ZNF506 in ZNF506-knockout U2OS cells (Supplementary Fig. 3f–h). Taken together, our data suggest that ZNF506 is a key player in the DNA double-strand break repair pathway through its interaction with MDC1.

It is apparent from our results so far that ZNF506 modulates the DNA double-strand break repair pathway somewhere between H2AX and MDC1. MDC1 is recruited by H2AX to the sites of DNA damage through a direct interaction between its BRCT domains and pS139 of H2AX^7^. We found that ZNF506 did not affect H2AX S139 phosphorylation (γH2AX, Fig. 1). Therefore, ZNF506 might regulate MDC1 focus formation through other mechanisms. It has been reported that H2AX is phosphorylated at the basal level (without DNA damage induction) at tyrosine 142 (Y142) by Williams–Beuren syndrome transcription factor (WSTF)^5^. Upon DNA damage, ATM phosphorylates the Ser139 residue while EYA (EYA1/ EYA3), a phosphatase, removes the phosphate group at Y142^6^. This allows for the interaction between MDC1 and H2AX, promoting signal amplification. To test whether ZNF506 plays a role in this interplay, we examined the effect of ZNF506 knockdown on H2AX pY142. Consistent with the earlier report, DNA damage induced Y142 dephosphorylation of H2AX (Fig. 3b). Loss of either ZNF506 or EYA1 blocked H2AX dephosphorylation, suggesting that ZNF506 regulates H2AX Y142 phosphorylation (Fig. 3b).

We next explored how ZNF506 regulates H2AX Y142 phosphorylation. Interestingly, we found a DNA damage-induced interaction between ZNF506 and EYA1. As shown in Fig. 3c, a robust interaction between the proteins was observed upon DNA damage. It had also been reported that EYA1 localizes to DNA double-strand break sites upon DNA damage^6^, although the underlying mechanism is still not clear. It is possible that ZNF506 might promote EYA1 recruitment. Indeed, we observed that EYA1 was unable to redistribute to DNA damage loci when ZNF506 was knocked down using shRNAs in U2OS cells (Fig. 3d, e and Supplementary Fig. 3i). It has been previously reported that phosphorylation of EYA3 at S219 residue is critical for its localization to the DNA damage site^6^. Indeed, mutation of the S219 residue of EYA3 to S219A abolished its interaction with ZNF506 (Fig. 3f). Therefore, ZNF506 is critical for the recruitment of EYA1/3 to DNA double-strand break sites and subsequent dephosphorylation of H2AX at Y142 residue to facilitate MDC1 recruitment, forming a positive feedback loop to facilitate mobilization of downstream repair factors and amplification of the repair signals.

We also investigated how ZNF506 binds EYA1/3. ZNF506 has one Krüppel-associated box (KRAB) and eight zinc-finger domains (Supplementary Fig. 3j). To identify the domain(s) critical for its role in DNA damage response, the indicated deletion mutants were generated. As shown in Fig. 3g, deletion of the zinc-finger domains (∆ZN) abrogated the DNA damage-induced interaction between ZNF506 and EYA1. In accordance with these data, a defect in DNA damage-mediated H2AX dephosphorylation was observed with the zinc-finger deletion mutant (∆ZN, Fig. 3h). Based on our data that MDC1 binds ZNF506 and ZNF506 binds EYA1, one can predict that MDC1 could interact with EYA1 through ZNF506. Indeed, in ZNF506-knockout cells, there was no MDC1-EYA1 interaction (Fig. 3i). In cells reconstituted with WT ZNF506, there was a robust DNA damage-induced MDC1-EYA1 interaction. However, in cells reconstituted with ZNF506 T140A, which disrupts the MDC1-ZNF506 interaction, the MDC1-EYA1 interaction was abolished (Fig. 3i), suggesting that ZNF506 mediates the MDC1-EYA interaction. These results establish ZNF506 as a mediator protein to facilitate EYA recruitment to DNA damage sites and H2AX Y142 dephosphorylation.

### ZNF506 is required for the maintenance of genomic stability

**Fig. 4:**
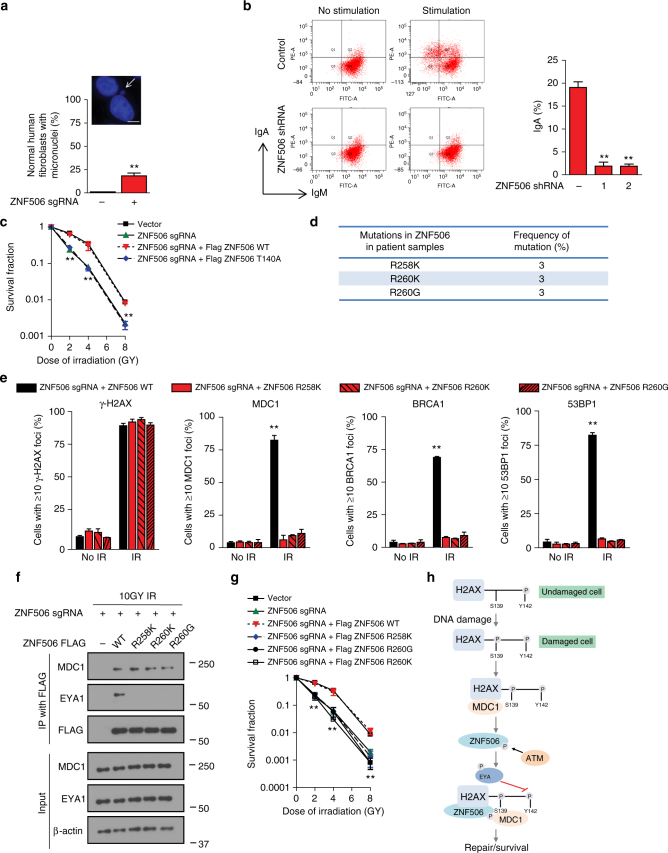
ZNF506 is required for the maintenance of genomic stability. **a** Loss of ZNF506 leads to genomic aberrations as indicated by micronuclei formation. ZNF506 was knocked out in normal human fibroblasts using CRISPR. Cells were stained with DAPI to assess micronuclei formation. Inset, representative image of cell with micronucleus. **b** ZNF506 is required for class switch recombination. Shown is a representative image of IgM to IgA conversion with treatment (left panel). **c** Loss of ZNF506 or lack of ZNF506 phosphorylation sensitizes cells to radiation. **d** Mutations in T-PLL identified in our patient sample. **e** The mutations in ZNF506 that were identified in patient samples lead to aberrant DNA damage response. **f** The patient mimetic mutations of ZNF506 disrupts the interaction between EYA1 and ZNF506 but not MDC1 and ZNF506. **g** The patient mimetic mutations of ZNF506 sensitizes cells to irradiation (IR). **h** Model for the role of ZNF506 in response to DNA double-strand break. We propose that ATM-mediated phosphorylation of ZNF506 promotes its interaction with MDC1 and recruits it to the DNA double-strand break site. It subsequently recruits the phosphatase EYA to the damage site. EYA, in turn, dephosphorylates H2AX at Y142 to recruit downstream repair factors and gradually amplify the DNA damage response for proper DNA double-strand break signaling. Shown are the representative data (mean ± SEM) from three independent experiments in **a**–**c**, **e**, and **g**. **p < 0.01 by ANOVA for control vs. each shRNA in **b**. **p < 0.01 by ANOVA for vector and ZNF506 sgRNA + FLAG ZNF506 WT vs. all other groups at each dose of IR in **c**. **p < 0.01 by ANOVA for ZNF506 sgRNA + FLAG ZNF506 WT vs. all other groups with IR in **e**. **p < 0.01 by ANOVA for vector and ZNF506 sgRNA + FLAG ZNF506 WT vs. all other groups at each dose of IR in **g**. Representative western blots in **f** are provided from three biologically independent experiments. Unprocessed blots are provided in Supplementary Fig. 5

If our hypothesis that ZNF506 regulates H2AX modification upon DNA damage is correct, we expect cells with aberrant or nonfunctional ZNF506 to demonstrate significant genomic instability. To assess if that in fact is true, we knocked out ZNF506 in normal human fibroblasts and assessed the number of cells displaying micronuclei formation, a marker of genomic instability^37^. We observed a drastic increase in micronuclei formation with ZNF506 loss, suggesting that the protein is important for the maintenance of genomic integrity (Fig. 4a and Supplementary Fig. 4a). Since ZNF506 is aberrant in leukemia, we wanted to assess the role of ZNF506 in class switch recombination. As shown in Fig. 4b and Supplementary Fig. 4b, downregulation of ZNF506 with shRNAs drastically reduced recombination. This result further substantiates the claim that ZNF506 plays a physiologic role and is important for DNA double-strand break repair. Furthermore, cells with ZNF506 knocked out or expressing the phosphorylation deficient ZNF506 T140A mutant were also hypersensitive to radiation, supporting that ZNF506 is important for proper DNA repair (Fig. 4c and Supplementary Fig. 4c).

Analysis of the TCGA database revealed that mutations in ZNF506 are frequently observed in various malignancies. In fact, aberration in ZNF506-EYA-H2AX axis is observed in up to 50% of some malignancies (TCGA Research Network: http://cancergenome.nih.gov/), suggesting that this pathway plays a critical role in the maintenance of genomic integrity. Of the various mutations, R258 and R260 localized in the zinc-finger domain were mutated in both our T-PLL patient samples as well as lung adenocarcinoma samples (TCGA Research Network: http://cancergenome.nih.gov/, Fig. 4d). To assess the physiologic relevance of these mutations, we constructed patient mimetic mutants and assessed the effect of these mutations on DNA damage response by assaying for γ-H2AX, MDC1, 53BP1, and BRCA1 foci formation. As shown in Fig. 4e and Supplementary Fig. 4d–g, the initial formation of γ-H2AX was unaffected by these mutations. However, foci formation of MDC1 and downstream repair proteins were drastically reduced. In U2OS cells knocked out for ZNF506 and subsequently reconstituted with either wild-type or the phosphorylation mutant (T140A), as expected, wild-type ZNF506 was able to rescue the foci formation of these critical DNA repair proteins, while the ZNF506 T140A was unable to do so (Supplementary Fig. 4d–f). To explore the mechanism of this observed defect, co-immunoprecipitation experiment with the various ZNF506 patient mimetic mutants was performed in irradiated HEK293 cells in which endogenous ZNF506 had been knocked out. Interestingly, the mutations in ZNF506 disrupted its interaction with EYA. However, the interaction with MDC1 was unaffected (Fig. 4f). Collectively, this suggests that the patient mimetic mutations block efficient DNA double-strand break repair, which can subsequently lead to genomic instability. Finally, due to the defects in DNA double-strand break repair observed with either loss or mutations in ZNF506, we expect cells harboring these mutations to be sensitive to DNA-damaging agents such as radiation. Indeed, cells mimicking the mutations found in patients were sensitive to radiation (Fig. 4g). Consistently, the ZNF506 gene is frequently dysregulated in various cancers including lung and breast cancers, where low ZNF506 mRNA levels in breast and lung cancers correlated with lower survival probability and worse prognosis (Supplementary Fig. 4h–i)^38,39^. This is consistent with the notion that genomic instability is often associated with poor prognosis.

## Discussion

DNA double-strand break repair and chromatin/histone modifications are intricately linked. Histone modifying enzymes play an important role in DNA damage response since histone tails and core domains demonstrate DNA damage-induced modifications such as phosphorylation and ubiquitylation. Regulation of these processes is important for proper DNA damage response and signal amplification. In this current study, we highlight a new regulatory mechanism controlling histone H2AX function. It is believed that the Y142 residue of H2AX is phosphorylated under basal conditions by WSTF^5^. In response to DNA damage, H2AX is phosphorylated at S139 by the protein kinases ATM/ATR^4^. This modification is associated with concurrent progressive dephosphorylation of Y142 by the protein phosphatase EYA^6,40^. However, it is still unclear what factor(s) determine the temporal switch from the diphosphorylated to the monophosphorylated state. Here we identified a new protein ZNF506 that regulates the modification of one of the earliest events occurring at DNA double-strand breaks, the gradual dephosphorylation of H2AX at Y142 by the phosphatase, EYA. This step is required for the recruitment of downstream repair proteins and amplification of the repair signal. Our results imply a positive feedback loop between MDC1 and ZNF506, which helps amplify the DNA damage response signal. In addition, ZNF506 deficiency leads to obvious genomic instability. For instance, similar to H2AX deficiency, lack of ZNF506 leads to defective class switch recombination. Search of cancer databases such as the TCGA supports a vital role of ZNF506 in tumor suppression and maintenance of genomic stability.

Zinc-finger proteins have been previously associated with DNA damage response. For instance, KRAB-associated protein 1 (KAP1) is a transcriptional co-repressor that regulates non-homologous end-joining-mediated DNA double-strand break repair^41,42^. In response to double-strand breaks, KAP1 is phosphorylated by ATM. This induces changes in chromatin conformation leading to chromatin decondensation and allows for the accrual of proteins in DNA damage response. Similarly, promyelocytic leukemia (PML)-Associated Repressor Of Transcription (PAROT) possesses both KRAB and zinc-finger domains and associates with heterochromatin protein 1 (HP1) family of proteins to induce transcriptional repression^43^. Like many proteins involved in DNA damage response, PAROT is present within the PML nuclear bodies (PML-NBs). Since PML-NBs have been shown to be tumor suppressors and regulate DNA repair, PAROT may have a similar function, though this has not been validated yet. Another example of proteins possessing zinc-finger domains is the poly (ADP-ribose) polymerase (PARP) family of proteins. The role of PARP1 in DNA damage response has been well studied and the protein has been extensively exploited for cancer therapy^44–47^. Protein poly ADP-ribosylation (PARylation), a widespread post-translational modification at DNA lesions catalyzed by PARP proteins, regulates a number of biological processes including chromatin reorganization, DNA damage response, transcriptional regulation, apoptosis, and mitosis. PARP1 recognizes DNA damage through its N-terminal DNA-binding domain, which consists of a tandem repeat of zinc-finger domain. Zinc-finger domains from separate PARP1 molecules form a strand break recognition module that activates PARP1 by facilitating its dimerization and subsequent trans-automodification^48^. Another example is the ubiquitin ligase CHFR which possesses a conserved putative C2H2 zinc-finger motif at its carboxy terminus and is important for the initial ubiquitylation at DNA damage sites^49,50^. As can be seen from these examples, even though zinc-finger proteins have similar structures, they are involved in a variety of roles. As such, ZNF506 may have roles other than the one outlined in this study and further studies are warranted to identify these functions.

EYA is a class of highly conserved proteins. Four family members have been identified so far: EYA1, EYA2, EYA3, and EYA4. The EYA family of tyrosine phosphatases has been shown to be required for cell survival and differentiation^51^. Loss-of-function mutations in EYA1 leads to branchio-oto-renal syndrome, further suggesting that the gene is important for normal cellular function^52^. EYA is the first transcriptional activator identified that harbor intrinsic phosphatase activity^53^. One of the lingering questions in the field is what dictates the distinct roles of this family of phosphatases. Recent breakthrough in understanding EYA function is the finding that EYA1 and EYA3 play vital roles in DNA repair vs. cell death decision after DNA damage^6,40^. Interestingly, the two phosphatases cannot compensate for the loss of the other^6,40^. So far, three substrates have been identified for EYA: H2AX, the atypical protein kinase C zeta, and the estrogen receptor-beta^6,54–57^. Increased EYA activity has been associated with tumor cell migration, invasion, and transformation^58^. However, detailed studies of the role of EYA in oncogenesis are lacking. Given that EYA has complex roles with its functions in transcriptional regulation and tyrosine dephosphorylation, it is likely a combination of activities that leads to cancer development.

Prior to our study, it was known that H2AX is phosphorylated at its Y142 residue by WSTF and the phosphatase EYA is required for its dephosphorylation. This dephosphorylation is important for the proper function of H2AX in recruiting MDC1 to facilitate DNA repair. Failure to remove the phosphate group compromises MDC1 recruitment and DNA repair, pushing the cells towards apoptosis as the dephosphorylated H2AX recruits the c-Jun N-terminal protein kinase 1 (JNK1) kinase^6^. ATM-mediated phosphorylation of EYA3 at S219 is required for its recruitment to the sites of DNA damage^6^. It was unclear what dictates this early event in the DNA damage response and how EYA localizes to the DNA lesion. Our study reveals how a zinc-finger protein, ZNF506 binds phosphorylated EYA and helps EYA recruitment. ZNF506 itself is also modified by ATM and is recruited to DNA damage sites through its interaction with MDC1. EYA in turn dephosphorylates H2AX Y142, further enhancing the recruitment of MDC1, ZNF506, and EYA. Therefore, the H2AX–MDC1–ZNF506-EYA axis forms a positive feedback loop to amplify γH2AX signaling.

We identified ZNF506 as a gene that is mutated in T-PLL. We do not have follow-up and survival data of patients with this extremely rare disease. However, a search of publicly available databases suggests that high expression of ZNF506 portends superior survival in lung and breast cancers (Supplementary Fig. 4h, i). As such, ZNF506 expression levels may have prognostic significance in malignancies including breast and lung cancers. In our study, we identified cancer-derived ZNF506 mutations to be defective in recruitment of DNA repair proteins and confer sensitivity to DNA-damaging agents. This puts forth the idea that DNA-damaging agents such as radiation may be a therapeutic option in patients with mutations in ZNF506.

In conclusion, our findings suggest an integrated model for how histone H2AX is regulated upon DNA damage to maintain genomic integrity. We report a new function of ZNF506 in the DNA damage response. We suggest that ZNF506 is a key component in the γH2AX signaling, and it helps recruit EYA and forms a H2AX–MDC1–ZNF506-EYA feedback loop in amplifying the DNA damage response repair signal (Fig. 4h). Mutations in ZNF506 are pathological and seen in cancer. We suggest that patients with mutations in ZNF506 that abolish its function may benefit from treatment with DNA-damaging agents such as radiation. Our results thus identify a key DNA damage-induced regulation of histone H2AX, which improves our understanding of this DNA repair pathway and allows for the design of better therapies for patients harboring mutations in this pathway.

## Methods

### Cell culture

The following cell lines were purchased from ATCC: HEK293, HEK293T, U2OS, and MDA-MB-231. The CH12F3-2a cell line was purchased from Thermo Fisher Scientific. The human fibroblast cell line utilized in this study has been described previously^59^. Cell lines were maintained in Dulbecco's modified Eagle's medium, McCoy’s 5A, or RPMI1640 with 10% fetal bovine serum (FBS) as recommended by the manufacturer. All cell lines were verified using short tandem repeat profiling by the Medical Genome facility at Mayo Clinic Center (Rochester, MN, USA). U2OS I-SceI cells were maintained in McCoy’s 5A supplemented with 10% FBS (kindly provided by Dr. Maria Jasin, Memorial Sloan Kettering Cancer Center, NY, USA).

### Chemicals

Triamcinolone acetonide and the ATM inhibitor KU55933 (Sigma) were used in this study.

### Plasmids

LentiCRISPR v2 (Plasmid #52961, provided by Dr. Feng Zhang) was purchased from Addgene. ZNF506, EYA1, and EYA3 complementary DNAs were purchased from Genscript and Dharmacon. ZNF506 was subcloned into pGEX-4T-2 vector (Clontech), CMV-FLAG, and peGFP vectors. All ZNF506 and EYA mutants were generated by site-directed mutagenesis (Stratagene). The MDC1 plasmids have been described previously^8,29,31,60^.

### Antibodies

Anti-BRCA1 (catalog #sc-6954, 1:200 dilution) and anti-EYA (catalog #sc-33605, 1:500 dilution) antibodies were purchased from Santa Cruz Biotechnology. Anti-γH2AX (catalog #05-636, 1:500 dilution), anti-53BP1 (catalog #05-725, 1:1000 dilution), anti-MDC1 (catalog #05-1572, 1:1000 dilution), and anti-RNF8 (catalog #09-813, 1:250 dilution) antibodies were purchased from Millipore. Anti-SQ/TQ (catalog #2851, 1:1000 dilution) and anti-GST (catalog #2625, 1:1000 dilution) antibodies were purchased from Cell Signaling. Anti-FLAG (catalog #F1804, 1:500 dilution), anti-HA (catalog #H6908, 1:500 dilution), and anti-β-actin (catalog #A2228, 1:10,000 dilution) antibodies were purchased from Sigma. Anti-RNF8 (catalog #ab4183, 1:200 dilution), anti-ZNF506 (catalog #ab179722, 1:250 dilution) and anti-EYA1 (catalog #ab85009, 1:250 dilution) antibodies were purchased from Abcam. Anti-ZNF506 antibody was purchased from Lifespan Biosciences (catalog #LS-C160889, 1:500 dilution). Anti-RNF8 antibody (1:500 dilution) was kindly provided by Dr. Xiaochun Yu (City of Hope, CA, USA). The following secondary antibodies were used as applicable to prevent the detection of immunoglobulin G (IgG) light or heavy chains: anti-mouse IgG VeriBlot for IP secondary antibody (Abcam, catalog #ab131368, 1:1000 dilution), IgG VeriBlot for IP secondary antibody (Abcam, catalog #ab131366, 1:200 dilution), mouse anti-rabbit IgG light-chain (Abcam, catalog #ab99697, 1:1000 dilution), peroxidase-AffiniPure goat anti-mouse IgG light-chain-specific (Jackson Immunoresearch, catalog #115-035-174, 1:2000 dilution), peroxidase IgG fraction monoclonal mouse anti-Rabbit IgG, light-chain-specific (Jackson Immunoresearch, catalog #211-032-171, 1:2000 dilution), or mouse anti-rabbit IgG (conformation specific, L27A9, Cell Signaling, catalog #3678, 1:2000 dilution).

### shRNA knockdown, CRISPR knockout, and peptides

Lentiviruses for infection of cells was performed as described previously^9^. ZNF506 and MDC1 CRISPR plasmids were generated using the methods described previously^61^. The following guides were used: ZNF506: CACATTTGTAGGGTTTCTCT and CGGTGTAAGCAGTGTAAACA; MDC1: GGTGTAACGTGGAGCCAGTAGGG and GGCCTACCACATTCTTCCCGAGG. The following peptides were synthesized and used in this manuscript: GLKQCLATTQRKIFQCDEY{Lys(Biotin)} and GLKQCLAT{pThr}QRKIFQCDEY{Lys(Biotin)} (GenScript).

### Immunoprecipitation, immunoblotting, and in vitro assay

We prepared cell lysates and performed immunoprecipitation and immunoblotting as previously described^9^. NETN buffer (20 mM Tris-HCl, pH 8.0, 100 mM NaCl, 1 mM EDTA, and 0.5% Nonidet P-40) containing 10 mM NaF, 50 mM β-glycerophosphate, 1 mg ml^−1^ pepstatin A, and 1 mg ml^−1^ aprotinin was used for cell lysis. Lysates were centrifuged at 9600 × *g* for 15 min to remove cellular debris. Proteins of interest were immunoprecipitated by incubating lysates with 2 µg of antibody and protein A or protein G Sepharose beads (Amersham Biosciences) anywhere from 2 h to overnight at 4 °C. Immunocomplexes were washed with NETN buffer, separated by sodium dodecyl sulfate-polyacrylamide gel electrophoresis (SDS-PAGE), and probed as indicated. Standard protocols were followed for immunoblotting. For assessment of interactions using GST-tagged proteins, GST fusion proteins were bound to glutathione Sepharose for 3 h at 4 °C, beads were rinsed twice with phosphate-buffered saline (PBS), incubated with indicated proteins for 1 h at 4 °C, washed with NETN buffer, boiled in loading buffer, and bound proteins were separated by SDS-PAGE. Blots were probed with the indicated antibodies.

### Recombinant protein production

Recombinant protein was produced using the method outlined previously^9^. The *Escherichia coli* strain *BL21* was grown in LB medium and used to express proteins. Isopropyl-β-d-thiogalactoside (0.25 mM) was used for induction (2 h at 37 °C followed by 12 h at 16 °C). Appropriate lysis buffer supplemented with Protease Inhibitor Cocktail (Roche) was used for cell lysis. Proteins were purified by binding to appropriate beads (3 h at 4 °C). Next, beads were rinsed twice with lysis buffer before the protein was finally eluted.

### Immunofluorescence

Immunostaining was performed as described previously^9^. The following protocol was used to assess ionizing radiation induced foci: cells were cultured on coverslips and exposed to 2 Gy IR. An hour later, depending on the foci to be stained, one of the following protocols were followed: (1) cells were rinsed with PBS, pre-extracted with a solution of 20 mM HEPES, pH 7.4, 50 mM NaCl, 3 mM MgCl_2_, 300 mM sucrose, and 0.5% Triton-X for 10 min at room temperature followed by fixation with 3% paraformaldehyde for 15 min at room temperature; (2) fixed with 3% paraformaldehyde for 15 min at room temperature without a pre-extraction step; or (3) fixed with 1:1 mixture of acetone: methanol at −20 °C. Triton (0.5%) solution for 5 min at room temperature was used to permeabilize cells. Samples were blocked with 5% goat serum, incubated with primary antibody for 30 min, washed three times to remove non-specific binding, incubated with secondary antibody for 30 min, and stained with 4′,6-diamidino-2-phenylindole (DAPI) to visualize nuclear DNA. Nikon eclipse 80i fluorescence microscope or laser scanning microscope (Zeiss LSM 880) was used for image acquisition. At least >200 cells were assessed per condition in all experiments where foci formation was analyzed.

### Comet assay

Comet assay was performed according to the manufacturer’s protocol (Trevigen). Briefly, cells were treated as indicated and collected at the specified time points. They were then embedded in low melting point agarose, allowed to set in 4 °C, lysed under neutral conditions, and subjected to electrophoresis. DNA was stained with Sybr green (Invitrogen). Images were acquired using Zeiss microscope and mean tail moment was assessed using Image J with the Comet Score plugin.

### Colony formation assay

Colony formation was performed as described previously^9^. Cells (500–5000) were plated in triplicate in each well of 6-well plates. After 16 h, cells were exposed to ionizing radiation, and left for 10–14 days at 37 °C to allow colony formation. Colonies were stained with methylene blue and counted. Results were normalized to plating efficiencies.

### Class switch recombination

Class switch recombination was performed in CH12F3-2a cells as described previously^9,62^. Briefly, ZNF506 was knocked down using shRNAs. After 40 h, cells were stimulated with the following ligands: 1 ng ml^−1^ of recombinant human tumor growth factor-β_1_ (R&D Systems), 250 ng ml^−1^ recombinant murine CD40 ligand (PerproTech), and 10 ng ml^−1^ of recombinant murine interleukin-4 (R&D Systems). After 60 h, cells were collected and stained with the following antibodies: phycoerythrin-conjugated anti-murine IgA antibody (clone 11-44-2, eBiosciences, catalog #12-5994-82) for intracellular staining and fluorescein isothiocyanate-conjugated anti-murine IgM antibody (eBiosciences; catalog #11-5890-82) for assessment of membrane IgM expression. The following buffers were used: Cytofix/Cytoperm and Perm/Wash buffers (BD Biosciences, catalog #554714). Cells were then analyzed on a FACS Calibur (BD Biosciences) at the Mayo Clinic Flow Cytometry Core to assess class switch from IgM (IgM+/IgA−) to IgA (IgM−/IgA+). The data acquired were analyzed using the FlowJo software (TreeStar).

### Survival analysis of cancer patients

Kaplan–Meier survival curves were generated using the Kaplan–Meier Plotter website for breast and lung cancers (http://kmplot.com)^38,39^ and statistical significance was determined by the log–rank test.

### Statistics

Data in bar and line graphs are reported as mean ± SEM. Statistical analyses were performed in GraphPad Prism with either analysis of variance (ANOVA) or *t* test as applicable. More than 200 cells were counted per experiment as indicated. The following depicts statistical significance: *p < 0.05; **p < 0.01. All results reported in this study were successfully repeated at least three times.

### Data availability

Source data for western blots are provided in Supplementary Fig. 5. All data supporting the findings of this study are available from the corresponding author on reasonable request.

## Electronic supplementary material

Supplementary Information

## References

[1] Jackson SP, Bartek J (2009). The DNA-damage response in human biology and disease. Nature.

[2] Zhou BBS, Elledge SJ (2000). The DNA damage response: putting checkpoints in perspective. Nature.

[3] Jeggo PA, Pearl LH, Carr AM (2016). DNA repair, genome stability and cancer: a historical perspective. Nat. Rev. Cancer.

[4] Rogakou EP, Pilch DR, Orr AH, Ivanova VS, Bonner WM (1998). DNA double-stranded breaks induce histone H2AX phosphorylation on serine 139. J. Biol. Chem..

[5] Xiao A (2009). WSTF regulates the function of H2A.X via a novel tyrosine kinase activity. Nature.

[6] Cook PJ (2009). Tyrosine dephosphorylation of H2AX modulates apoptosis and survival decisions. Nature.

[7] Stucki M (2005). MDC1 directly binds phosphorylated histone H2AX to regulate cellular responses to DNA double-strand breaks. Cell.

[8] Lou Z (2006). MDC1 maintains genomic stability by participating in the amplification of ATM-dependent DNA damage signals. Mol. Cell.

[9] Nowsheen S (2018). L3MBTL2 orchestrates ubiquitin signalling by dictating the sequential recruitment of RNF8 and RNF168 after DNA damage. Nat. Cell Biol..

[10] Mattiroli F (2012). RNF168 ubiquitinates K13-15 on H2A/H2AX to drive DNA damage signaling. Cell.

[11] Panier S (2012). Tandem protein interaction modules organize the ubiquitin-dependent response to DNA double-strand breaks. Mol. Cell.

[12] Doil C (2009). RNF168 binds and amplifies ubiquitin conjugates on damaged chromosomes to allow accumulation of repair proteins. Cell.

[13] Fradet-Turcotte A (2013). 53BP1 is a reader of the DNA-damage-induced H2A Lys 15 ubiquitin mark. Nature.

[14] Sobhian B (2007). RAP80 targets BRCA1 to specific ubiquitin structures at DNA damage sites. Science.

[15] Kim H, Chen J, Yu X (2007). Ubiquitin-binding protein RAP80 mediates BRCA1-dependent DNA damage response. Science.

[16] Wang B, Elledge SJ (2007). Ubc13/Rnf8 ubiquitin ligases control foci formation of the Rap80/Abraxas/Brca1/Brcc36 complex in response to DNA damage. Proc. Natl. Acad. Sci. USA.

[17] Powell SN, Kachnic LA (2003). Roles of BRCA1 and BRCA2 in homologous recombination, DNA replication fidelity and the cellular response to ionizing radiation. Oncogene.

[18] Pellegrini L (2002). Insights into DNA recombination from the structure of a RAD51–BRCA2 complex. Nature.

[19] Roy R, Chun J, Powell SN (2012). BRCA1 and BRCA2: different roles in a common pathway of genome protection. Nat. Rev. Cancer.

[20] Xu G (2015). REV7 counteracts DNA double-strand break resection and affects PARP inhibition. Nature.

[21] Drané P (2017). TIRR regulates 53BP1 by masking its histone methyl-lysine binding function. Nature.

[22] Chapman JR (2013). RIF1 is essential for 53BP1-dependent non-homologous end joining and suppression of DNA double-strand break resection. Mol. Cell.

[23] Escribano-Díaz C (2013). A cell cycle-dependent regulatory circuit composed of 53BP1-RIF1 and BRCA1-CtIP controls DNA repair pathway choice. Mol. Cell.

[24] Zhang H (2016). A cell cycle-dependent BRCA1–UHRF1 cascade regulates DNA double-strand break repair pathway choice. Nat. Commun..

[25] Hsi AC (2014). T-cell prolymphocytic leukemia frequently shows cutaneous involvement and is associated with gains of MYC, loss of ATM, and TCL1A rearrangement. Am. J. Surg. Pathol..

[26] Stilgenbauer S (1997). Biallelic mutations in the ATM gene in T-prolymphocytic leukemia. Nat. Med..

[27] Stoppa-Lyonnet D (1998). Inactivation of the ATM gene in T-cell prolymphocytic leukemias. Blood.

[28] Zeitlin SG (2009). Double-strand DNA breaks recruit the centromeric histone CENP-A. Proc. Natl. Acad. Sci. USA.

[29] Lou Z, Minter-Dykhouse K, Wu X, Chen J (2003). MDC1 is coupled to activated CHK2 in mammalian DNA damage response pathways. Nature.

[30] Goldberg M (2003). MDC1 is required for the intra-S-phase DNA damage checkpoint. Nature.

[31] Stewart GS, Wang B, Bignell CR, Taylor AMR, Elledge SJ (2003). MDC1 is a mediator of the mammalian DNA damage checkpoint. Nature.

[32] Rodriguez M, Yu X, Chen J, Songyang Z (2003). Phosphopeptide binding specificities of BRCA1 COOH-terminal (BRCT) domains. J. Biol. Chem..

[33] Yu X, Chini CC, He M, Mer G, Chen J (2003). The BRCT domain is a phospho-protein binding domain. Science.

[34] Kolas NK (2007). Orchestration of the DNA-damage response by the RNF8 ubiquitin ligase. Sci. (New York, NY).

[35] Kim ST, Lim DS, Canman CE, Kastan MB (1999). Substrate specificities and identification of putative substrates of ATM kinase family members. J. Biol. Chem..

[36] Bekker-Jensen S, Mailand N (2010). Assembly and function of DNA double-strand break repair foci in mammalian cells. DNA Repair (Amst.)..

[37] Crasta K (2012). DNA breaks and chromosome pulverization from errors in mitosis. Nature.

[38] Lanczky A (2016). miRpower: a web-tool to validate survival-associated miRNAs utilizing expression data from 2178 breast cancer patients. Breast Cancer Res. Treat..

[39] Győrffy B, Surowiak P, Budczies J, Lánczky A (2013). Online survival analysis software to assess the prognostic value of biomarkers using transcriptomic data in non-small-cell lung cancer. PLoS ONE.

[40] Krishnan N (2009). Dephosphorylation of the C-terminal tyrosyl residue of the DNA damage-related histone H2A.X is mediated by the protein phosphatase eyes absent. J. Biol. Chem..

[41] Ziv Y (2006). Chromatin relaxation in response to DNA double-strand breaks is modulated by a novel ATM-and KAP1 dependent pathway. Nat. Cell Biol..

[42] Lin YH (2015). KAP1 deacetylation by SIRT1 promotes non-homologous end-joining repair. PLoS ONE.

[43] Fleischer S, Wiemann S, Will H, Hofmann TG (2006). PML-associated repressor of transcription (PAROT), a novel KRAB-zinc-finger repressor, is regulated through association with PML nuclear bodies. Exp. Cell Res..

[44] Rouleau M, Patel A, Hendzel MJ, Kaufmann SH, Poirier GG (2010). PARP inhibition: PARP1 and beyond. Nat. Rev. Cancer.

[45] Lord CJ, Tutt AN, Ashworth A (2015). Synthetic lethality and cancer therapy: lessons learned from the development of PARP inhibitors. Annu. Rev. Med..

[46] Mullard, A. PARP inhibitors plough on. *Nat. Rev. Drug Discov.* **16**, 229 (2017). 10.1038/nrd.2017.61 28356598

[47] Jackson SP, Helleday T (2016). Drugging DNA repair. Science.

[48] Ali AAE (2012). The zinc-finger domains of PARP1 cooperate to recognise DNA strand breaks. Nat. Struct. Mol. Biol..

[49] Ahel I (2008). Poly(ADP-ribose)-binding zinc-finger motifs in DNA repair/checkpoint proteins. Nature.

[50] Liu C, Wu J, Paudyal SC, You Z, Yu X (2013). CHFR is important for the first wave of ubiquitination at DNA damage sites. Nucleic Acids Res..

[51] Bonini NM, Leiserson WM, Benzer S (1993). The eyes absent gene: genetic control of cell survival and differentiation in the developing Drosophila eye. Cell.

[52] Vincent C (1997). BOR and BO syndromes are allelic defects of EYA1. Eur. J. Hum. Genet..

[53] Li X (2003). Eya protein phosphatase activity regulates Six1–Dach–Eya transcriptional effects in mammalian organogenesis. Nature.

[54] El-Hashash AH (2011). Eya1 controls cell polarity, spindle orientation, cell fate and Notch signaling in distal embryonic lung epithelium. Development.

[55] Stokes MP (2007). Profiling of UV-induced ATM/ATR signaling pathways. Proc. Natl. Acad. Sci. USA.

[56] Tootle TL (2003). The transcription factor Eyes absent is a protein tyrosine phosphatase. Nature.

[57] Yuan B (2014). A phosphotyrosine switch determines the antitumor activity of ERβ. J. Clin. Invest..

[58] Pandey RN (2010). The Eyes Absent phosphatase-transactivator proteins promote proliferation, transformation, migration, and invasion of tumor cells. Oncogene.

[59] Kanakkanthara A (2016). Cyclin A2 is an RNA binding protein that controls Mre11 mRNA translation. Science.

[60] Lou Z, Chini CCS, Minter-Dykhouse K, Chen J (2003). Mediator of DNA damage checkpoint protein 1 regulates BRCA1 localization and phosphorylation in DNA damage checkpoint control. J. Biol. Chem..

[61] Sanjana NE, Shalem O, Zhang F (2014). Improved vectors and genome-wide libraries for CRISPR screening. Nat. Methods.

[62] Pei H (2013). The histone methyltransferase MMSET regulates class switch recombination. J. Immunol..

