# Supplementary material for: ZNF506-dependent positive feedback loop regulates H2AX signaling after DNA damage
Source: Nat Commun. 2018 Jul 16;9:2736. doi: 10.1038/s41467-018-05161-0 (PMC6048040; doi:10.1038/s41467-018-05161-0)
Supplement: Supplementary file 1 — Supplementary Information [file 41467_2018_5161_MOESM1_ESM.pdf]

Supplementary Information

ZNF506-dependent Positive Feedback Loop Regulates H2AX Signaling after DNA Damage

Somaira Nowsheen[1,2], Khaled Aziz[1], Kuntian Luo[3], Min Deng[3], Bo Qin[3], Jian Yuan[2,4], Karthik B Jeganathan[5], Jia Yu[2], Henan Zhang[6], Wei Ding[6], Jan M van Deursen[5] and Zhenkun Lou[2,3*]

[1]Mayo Clinic Medical Scientist Training Program, Mayo Clinic School of Medicine and Mayo Clinic Graduate School of Biomedical Sciences, Mayo Clinic, Rochester, MN 55905.

[2]Department of Molecular Pharmacology and Experimental Therapeutics, Mayo Clinic, Rochester, MN 55905, USA.

[3]Department of Oncology, Mayo Clinic, Rochester, MN 55905, USA.

[4]Research Center for Translational Medicine, Key Laboratory of Arrhythmias of the Ministry of Education of China, East Hospital, Tongji University School of Medicine, Shanghai 200120, China.

[5]Department of Pediatrics and Adolescent Medicine, Mayo Clinic, Rochester, MN 55905, USA.

[6]Department of Hematology, Mayo Clinic, Rochester, MN 55905, USA.

*Correspondence to: Zhenkun Lou (Lou. zhenkun@mayo.edu).

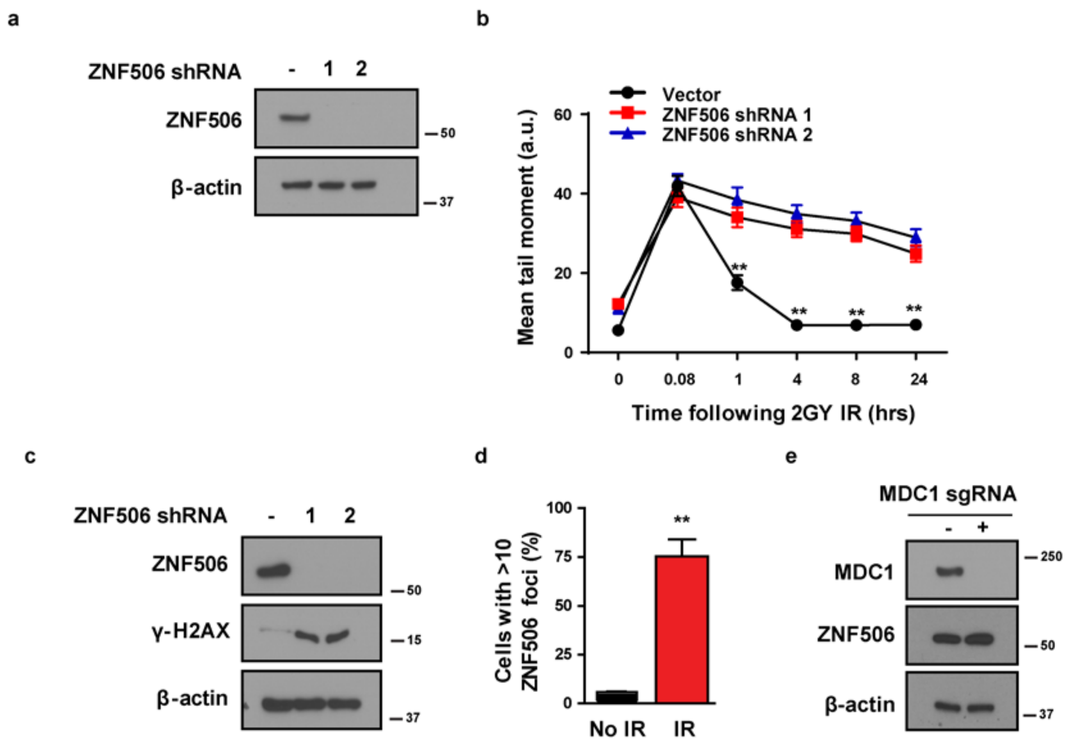

Supplementary Figure 1: ZNF506 regulates DNA damage response. a-b, Depletion of ZNF506 leads to DNA repair defect. **a,** Representative western blot showing knockdown efficiency of ZNF506 using shRNAs. **b,** Quantification of mean tail moment of cells subjected to comet assay after the indicated treatment. **c,** Representative western blot showing knockdown efficiency of ZNF506 using shRNAs (**related to Figure 1a-b**). **d,** Quantification of U2OS cells with ZNF506 foci (**related to Figure 1d**). **e,** Representative western blot showing the protein level of ZNF506 in MDC1 knockout U2OS cells (**related to Figure 1e-f**). Shown are the representative data (Mean ± S.E.M.) from three independent experiments in **b** and **d**. **p<0.01 by ANOVA for vector vs all other groups at each time point in **b**. **p<0.01 by t-test for no irradiation (IR) vs IR in **d**. Representative western blots in **a, c,** and **e** are provided from 3 biologically independent experiments. Unprocessed blots are provided in **Supplementary Figure 5**.

a

MGPLQFRDVAIEFSLEEWHCLDAAQRNLYRDVMLENYRNLIFLGIVVSKPNLITCLEQGKKPLTMKRHEMIAKPP
VMYSHFAQDLWSEQSIKDSFQKVILRRYEKCRHDNLQLKKGCESVDECPVHKRGYNGLKQCLAT<u>**TQ**</u>RKIFQCDE
YVKFLHKFSNSNKHKIRDTGKKSFKCIEYGKTFNQSSTRTTYKKIDAGEKRYKCEECGKAYKQSSHLTTHKKIHT
GEKPYKCEECGKAYKQSCNLTTHKIIHTGEKPYRCRECGKAFNHPATLFSHKKIHTGEKPYKCDKCGKAFISSSTL
TKHEIIHTGEKPYKCEECGKAFNRSSNLTKHKRIHTGDVPYKCDECGKTFTWYSSLSKHKRAHTGEKPYKCEECG
KAFTAFSTLTEHKIIHTGEKPYKCEECGKAFNWSSALNKHKKIHIRQKPCIVKNVENLLNVPQPLISIR

Supplementary Figure 2: a, Protein sequence of ZNF506. The putative ATM phosphorylation site is shown in red and underlined (**related to Figure 2c**).

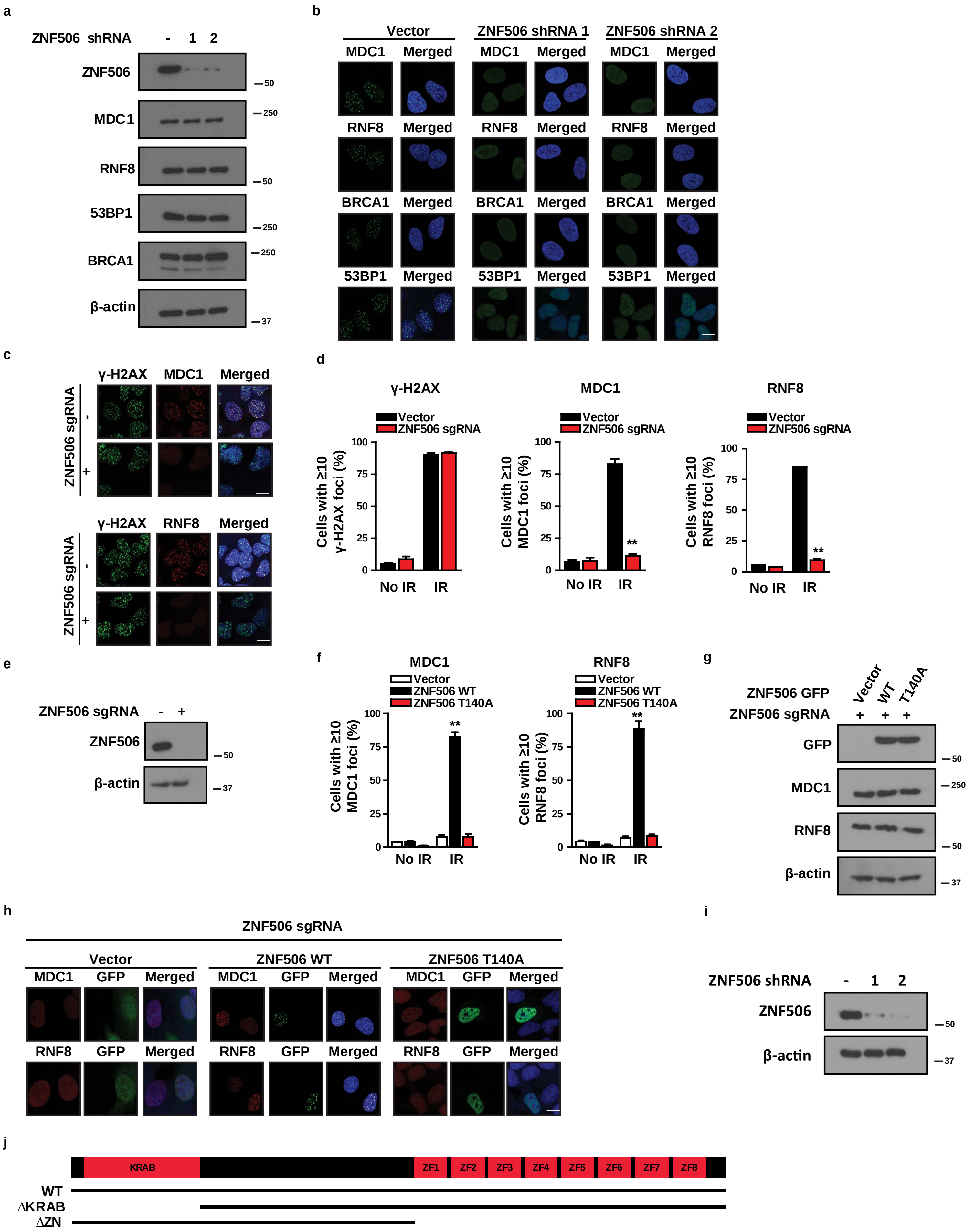
a
ZNF506 shRNA - 1 2
ZNF506
MDC1
RNF8
53BP1
BRCA1
β-actin
50
250
50
250
250
37
b
Vector
ZNF506 shRNA 1
ZNF506 shRNA 2
MDC1 Merged
RNF8 Merged
BRCA1 Merged
53BP1 Merged
c
γ-H2AX MDC1 Merged
γ-H2AX RNF8 Merged
ZNF506 sgRNA
-
+
d
γ-H2AX
MDC1
RNF8
Vector
ZNF506 sgRNA
Cells with ≥10 γ-H2AX foci (%)
Cells with ≥10 MDC1 foci (%)
Cells with ≥10 RNF8 foci (%)
No IR IR
**
e
ZNF506 sgRNA - +
ZNF506
β-actin
50
37
f
MDC1
RNF8
Vector
ZNF506 WT
ZNF506 T140A
Cells with ≥10 MDC1 foci (%)
Cells with ≥10 RNF8 foci (%)
No IR IR
**
g
ZNF506 GFP Vector WT T140A
ZNF506 sgRNA + + +
GFP
MDC1
RNF8
β-actin
50
250
50
37
h
ZNF506 sgRNA
Vector
ZNF506 WT
ZNF506 T140A
MDC1 GFP Merged
RNF8 GFP Merged
i
ZNF506 shRNA - 1 2
ZNF506
β-actin
50
37
j
KRAB
ZF1 ZF2 ZF3 ZF4 ZF5 ZF6 ZF7 ZF8
WT
ΔKRAB
ΔZN

Supplementary Figure 3: ZNF506 modulates DNA damage response. a-b, Knockdown of ZNF506 affects localization of DNA repair proteins to the DNA double strand break site (**related to Figure 3a**). **a,** Representative western blot showing the expression level of various DNA repair proteins is not altered with ZNF506 knockdown. **b,** Representative images of MDC1, RNF8, BRCA1 and 53BP1 foci in U2OS cells after the indicated treatments. Nucleus is stained with DAPI (blue). **c-e,** ZNF506 knockout affects localization of DNA repair proteins to the DNA double strand break site. **c,** Representative images of γ-H2AX, MDC1, and RNF8 foci in U2OS cells after the indicated treatments. Nucleus is stained with DAPI (blue). **d,** Quantification of γ-H2AX, MDC1, and RNF8 foci in U2OS cells after the indicated treatments. **e,** Representative western blot showing the ZNF506 knockout efficiency. **f-h,** Expression of wild-type (WT) but not the phosphorylation mutant (T140A) of ZNF506 (green) restores foci formation of DNA repair proteins. **f,** Quantification of MDC1 and RNF8 foci in U2OS cells after the indicated treatments. **g,** Representative western blot showing the expression level of the indicated plasmids and repair proteins. **h,** Representative images of MDC1 and RNF8 foci in U2OS cells after the indicated treatments. Nucleus is stained with DAPI (blue). **i,** Representative western blot showing the knockdown efficiency of the ZNF506 shRNAs (**related to Figure 3d-e**). **j,** Structure of ZNF506 (**related to Figure 3g-h**). ZNF506 has a KRAB and eight zinc finger (ZN) domains. Shown are the representative data (Mean ± S.E.M.) from three independent experiments in **d** and **f**. **$p<0.01$ by ANOVA for vector vs knockout with irradiation (IR) in **d**. **$p<0.01$ by ANOVA for ZNF506 WT vs all other groups with IR in **f**. Representative images of three independent experiments are shown in **b**, **c** and **h**. Scale bars, 10 μm. Representative western blots in **a, e, g,** and **i** are provided from 3 biologically independent experiments. Unprocessed blots are provided in **Supplementary Figure 5**.

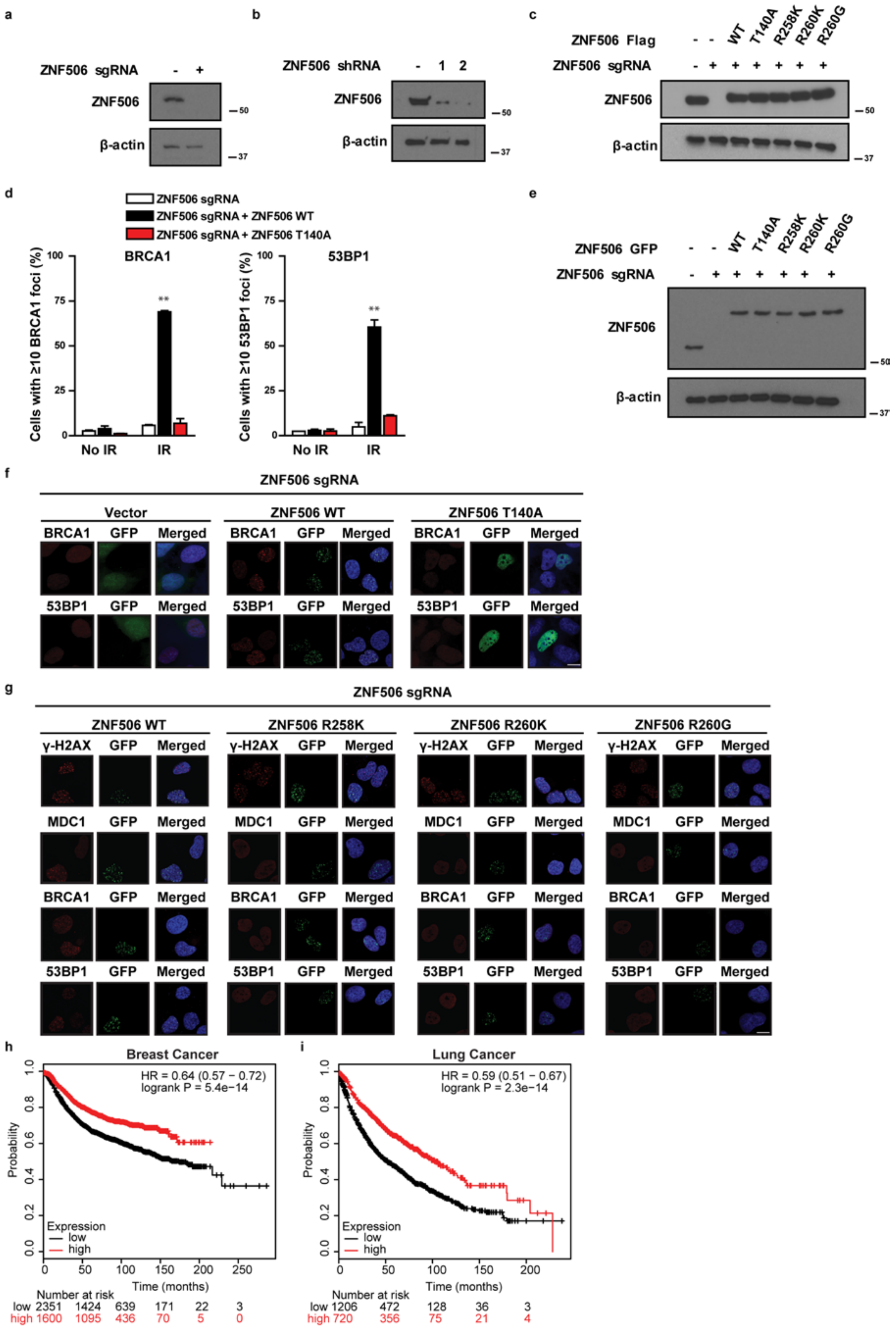
a
ZNF506 sgRNA - +
ZNF506
β-actin
50
37
b
ZNF506 shRNA - 1 2
ZNF506
β-actin
50
37
c
ZNF506 Flag - - WT T140A R258K R260K R260G
ZNF506 sgRNA - + + + + + +
ZNF506
β-actin
50
37
d
ZNF506 sgRNA
ZNF506 sgRNA + ZNF506 WT
ZNF506 sgRNA + ZNF506 T140A
BRCA1
Cells with ≥10 BRCA1 foci (%)
53BP1
Cells with ≥10 53BP1 foci (%)
100
75
50
25
0
**
No IR
IR
e
ZNF506 GFP - - WT T140A R258K R260K R260G
ZNF506 sgRNA - + + + + + +
ZNF506
β-actin
50
37
f
ZNF506 sgRNA
Vector
ZNF506 WT
ZNF506 T140A
BRCA1 GFP Merged
53BP1 GFP Merged
g
ZNF506 sgRNA
ZNF506 WT
ZNF506 R258K
ZNF506 R260K
ZNF506 R260G
γ-H2AX GFP Merged
MDC1 GFP Merged
BRCA1 GFP Merged
53BP1 GFP Merged
h
Breast Cancer
HR = 0.64 (0.57 − 0.72)
logrank P = 5.4e−14
Probability
Expression
low
high
Time (months)
0 50 100 150 200 250
0.0 0.2 0.4 0.6 0.8 1.0
Number at risk
low 2351 1424 639 171 22 3
high 1600 1095 436 70 5 0
i
Lung Cancer
HR = 0.59 (0.51 − 0.67)
logrank P = 2.3e−14
Probability
Expression
low
high
Time (months)
0 50 100 150 200
0.0 0.2 0.4 0.6 0.8 1.0
Number at risk
low 1206 472 128 36 3
high 720 356 75 21 4

Supplementary Figure 4: ZNF506 is required for the maintenance of genomic integrity. a, Representative western blot showing knockout efficiency of ZNF506 using sgRNA in human fibroblasts (**related to Figure 4a**). **b,** Representative western blot showing knockdown efficiency of ZNF506 using shRNA in CH12F3-2a cells (**related to Figure 4b**). **c,** Representative western blot showing the expression level of the indicated plasmids in MDA-MB-231 cells in which ZNF506 was knocked out using sgRNA (**related to Figures 4c and 4g**). **d-f,** Phosphorylation of ZNF506 is required for recruitment of BRCA1 and 53BP1 to DNA damage sites. Cells were stained for the indicated foci (red). Nucleus is stained with DAPI (blue). **d,** Quantification of the indicated foci in U2OS cells after the indicated treatment. **e,** Representative western blot showing the expression level of the indicated plasmids in U2OS cells in which ZNF506 was knocked out using sgRNA (**also related to Figure 4e**). **f,** Representative images of the indicated foci in U2OS cells treated as indicated and fixed an hour after 2GY irradiation (IR). **g,** The mutations in ZNF506 that were identified in patient samples lead to aberrant DNA damage response. Shown are representative images of the indicated foci in U2OS cells after the indicated treatment (**related to Figure 4e**). **h-i,** Kaplan–Meier analysis showing a tight correlation between ZNF506 expression levels and patient survival. Data were from breast and lung cancer patients with low and high ZNF506 expression. Patient number at risk at different times of analyses is indicated at the bottom of the plots. The plots were generated using the KmPlot tool (http://www.kmplot.com/lung and http://www.kmplot.com/breast). Shown are the representative data (Mean ± S.E.M.) from three independent experiments in **d**. $^{**}p<0.01$ by ANOVA for ZNF506 sgRNA+ZNF506 WT vs all other groups with IR in **d**. Representative images of three independent experiments are shown in **f-g**. Scale bars, 10 μm. Representative western blots in **a-c,** and **e** are provided from 3 biologically independent experiments. Unprocessed blots are provided in **Supplementary Figure 5**.

Figure 1g

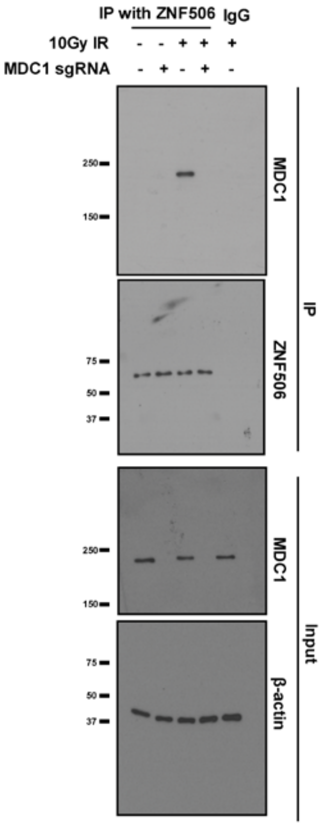

Figure 2a

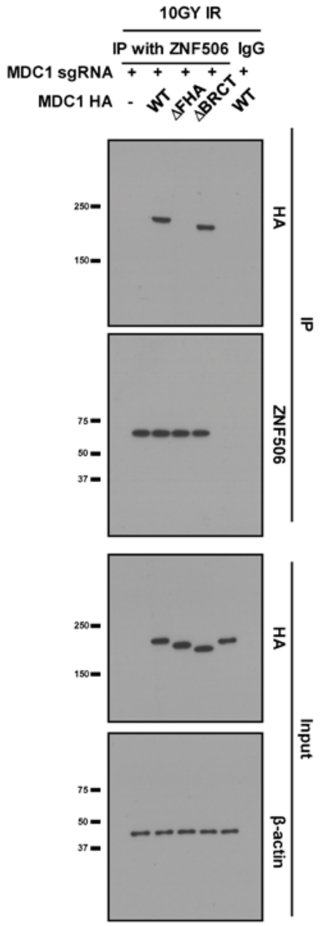

Figure 2b

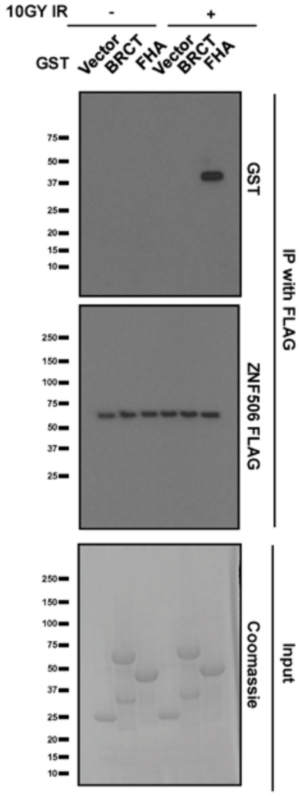

Figure 2c

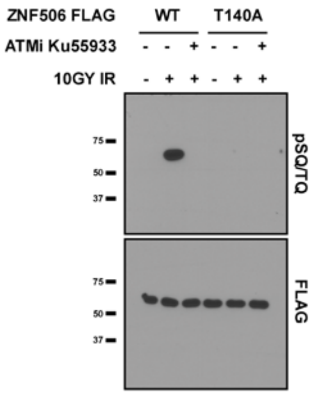

Figure 2e

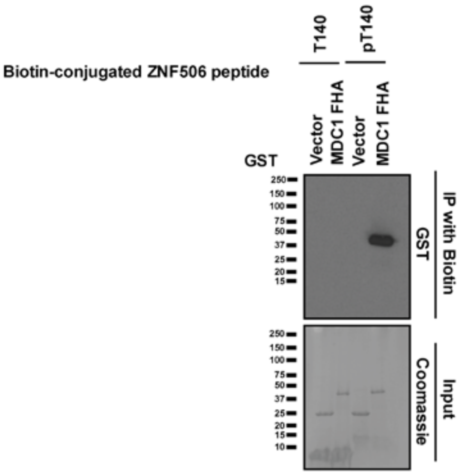

Supplementary Figure 5: Unprocessed images of all blots in this manuscript.

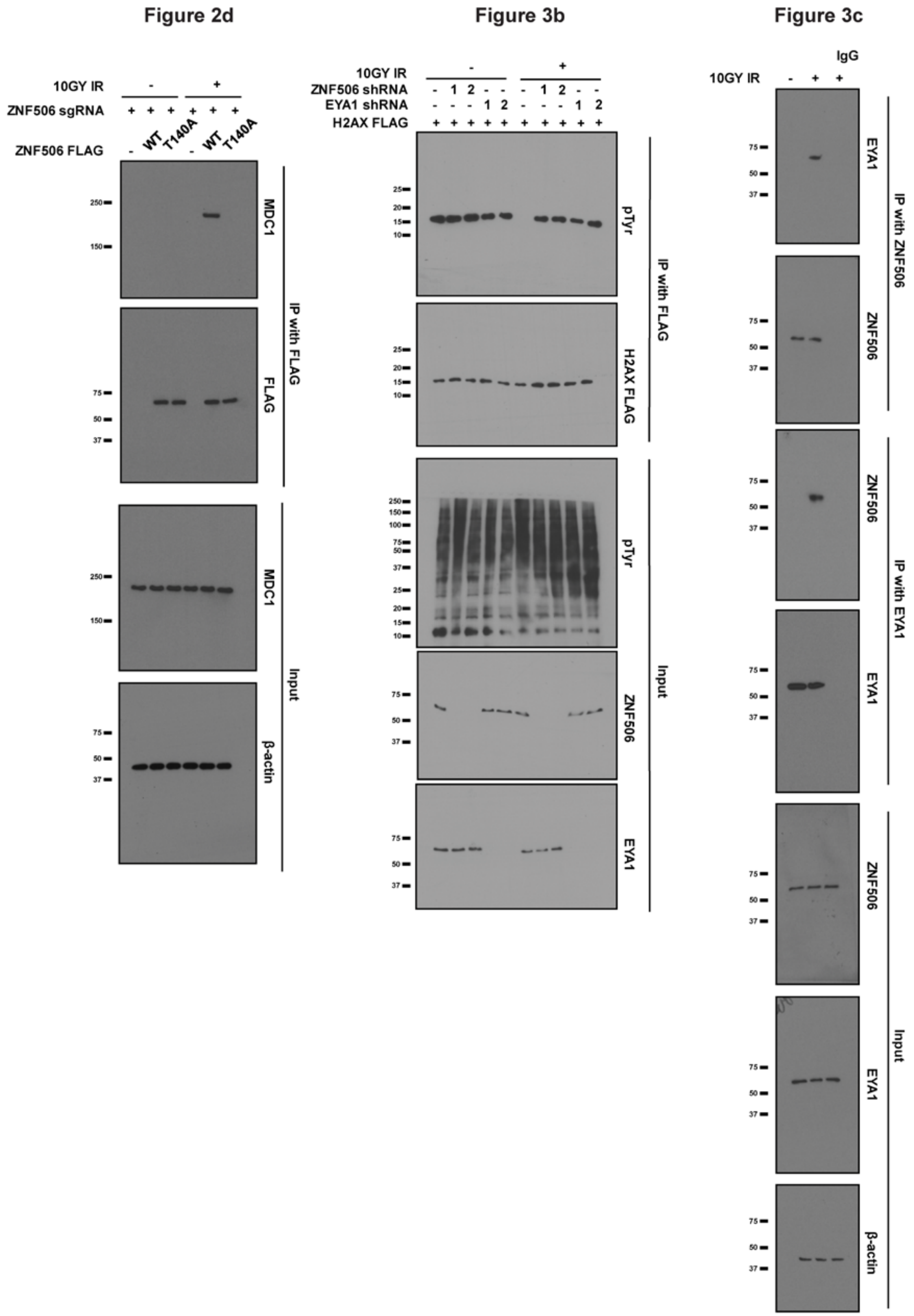

Supplementary Figure 5 continued: Unprocessed images of all blots in this manuscript.

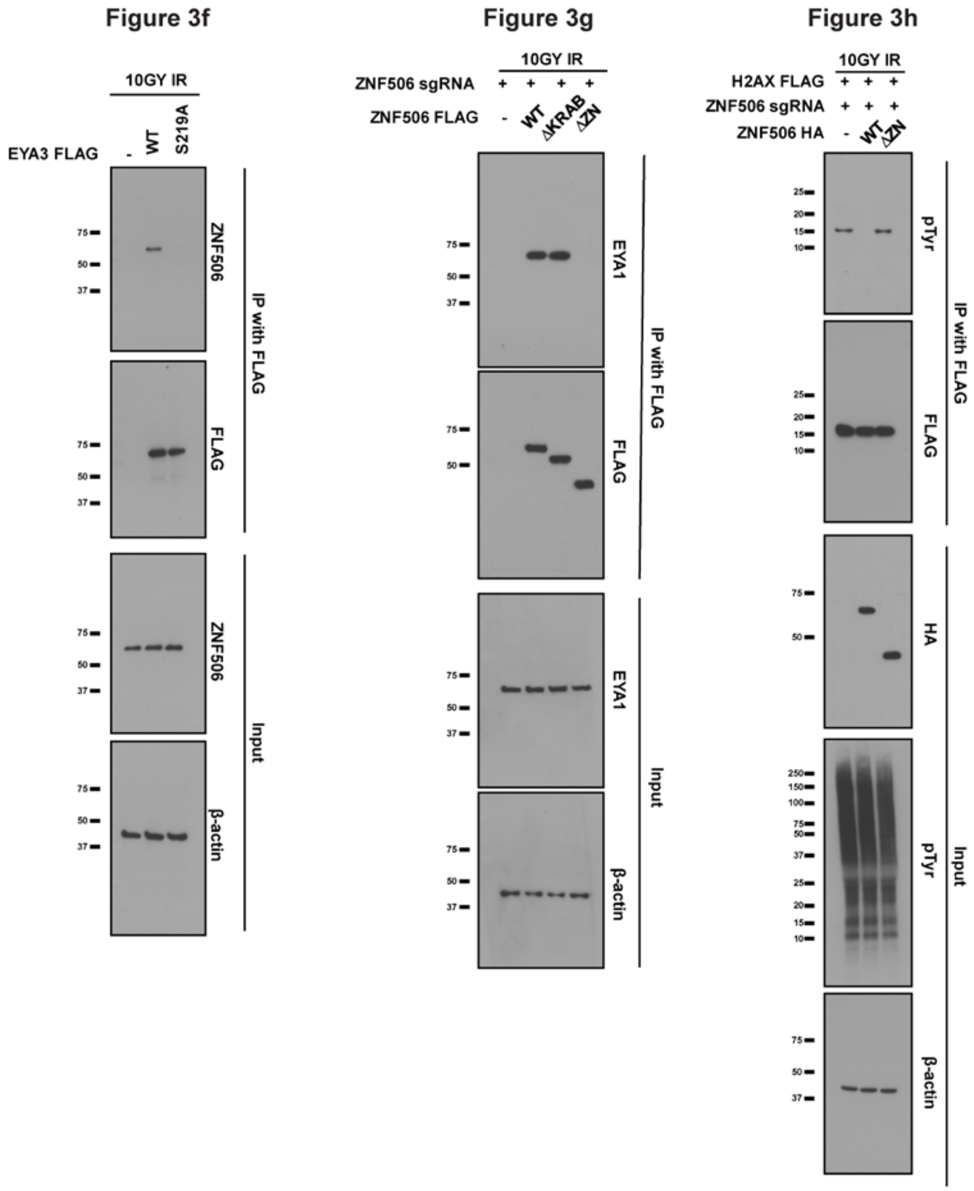

Supplementary Figure 5 continued: Unprocessed images of all blots in this manuscript.

Figure 3i

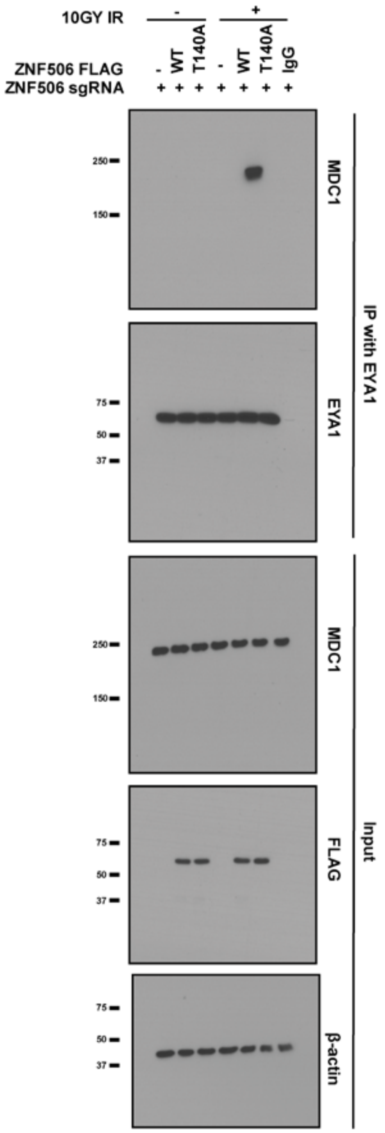

Figure 4f

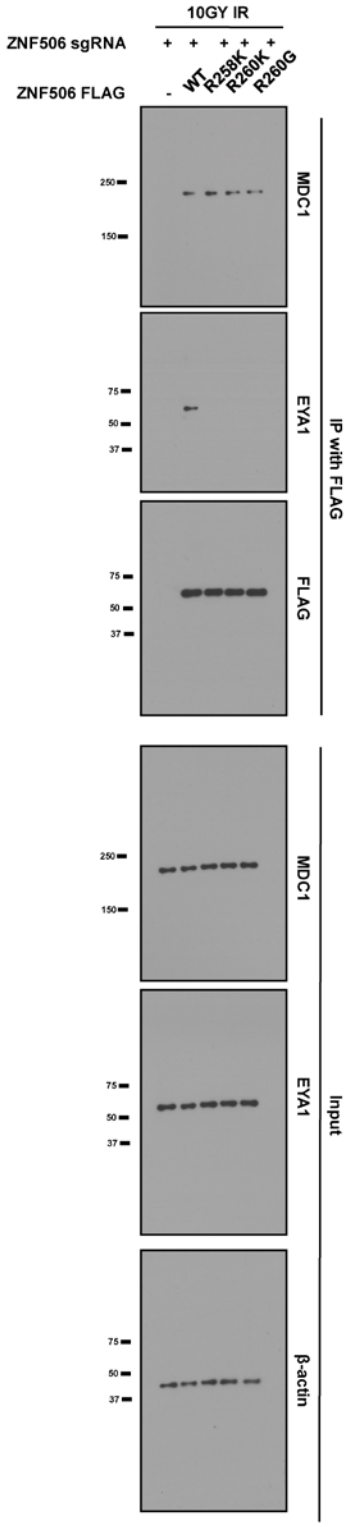

Supplementary Figure 1a

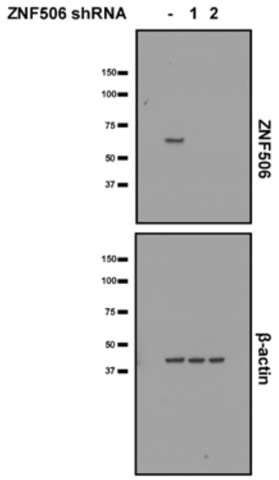

Supplementary Figure 1c

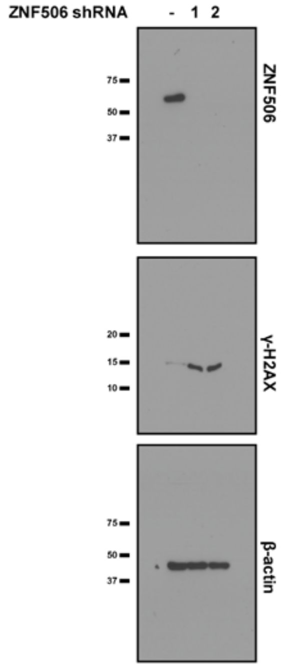

Supplementary Figure 5 continued: Unprocessed images of all blots in this manuscript.

Supplementary Figure 1e

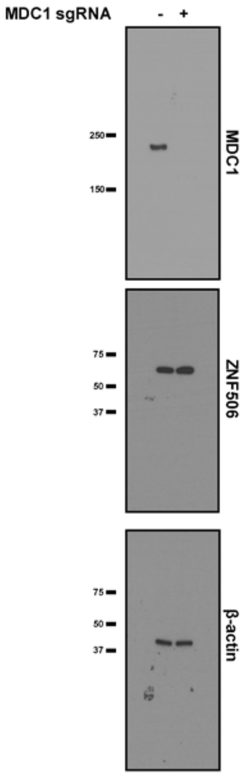

Supplementary Figure 3a

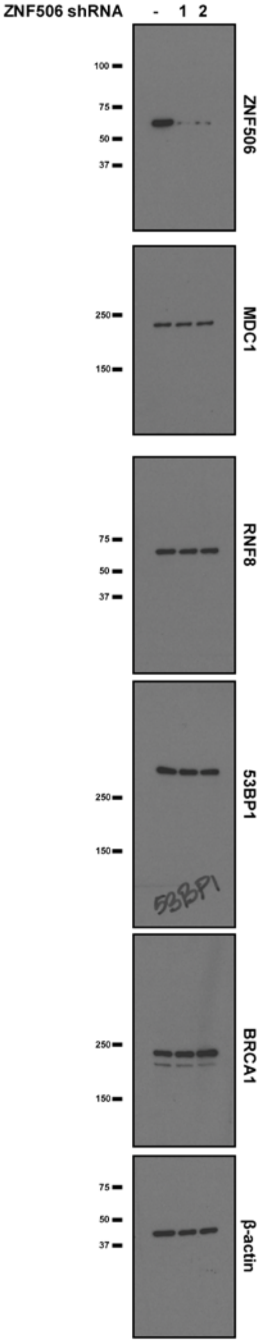

Supplementary Figure 3g

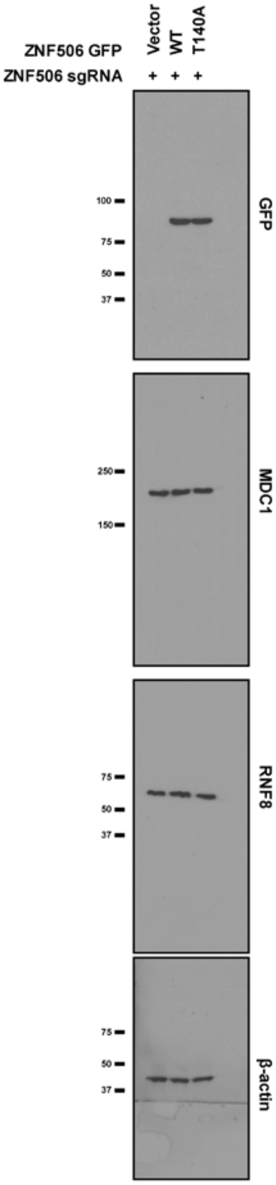

Supplementary Figure 3e

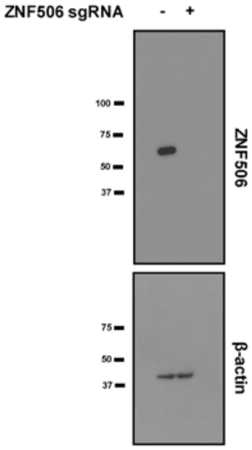

Supplementary Figure 5 continued: Unprocessed images of all blots in this manuscript.

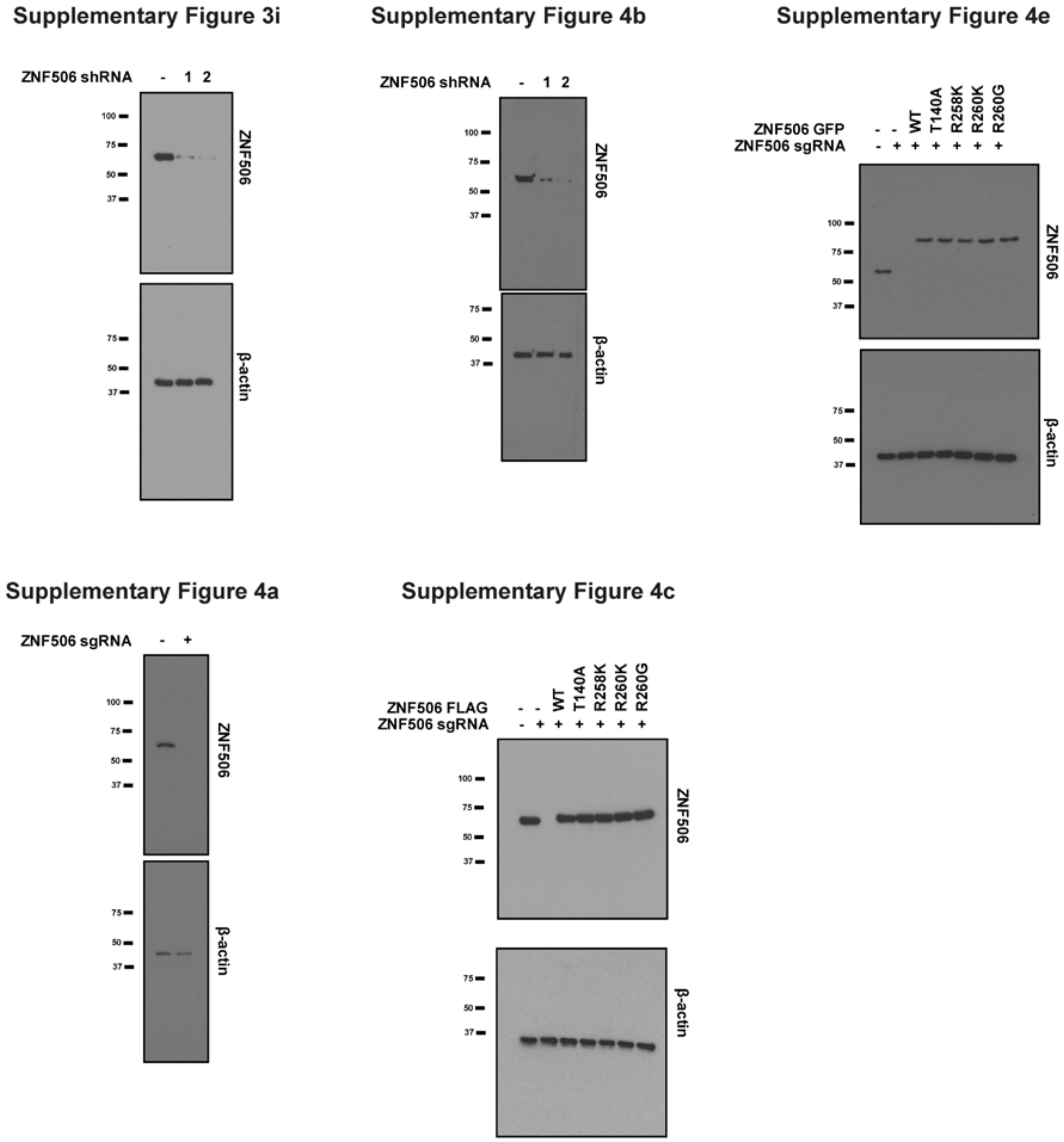

Supplementary Figure 5 continued: Unprocessed images of all blots in this manuscript.
